# Automated insulin delivery in pregnant women with type 1 diabetes mellitus: a systematic review and meta-analysis

**DOI:** 10.1007/s00592-025-02446-x

**Published:** 2025-01-10

**Authors:** Athina Stamati, Athanasios Christoforidis

**Affiliations:** 1 School of Medicine, Faculty of Health Science, Aristotle University of Thessaloniki, 106, Mitropoleos Str, 54621 Thessaloniki, Greece; 2 1st Paediatric Department, School of Medicine, Faculty of Health Sciences, Ippokratio General Hospital, Aristotle University of Thessaloniki, Thessaloniki, Greece

**Keywords:** Automated insulin delivery, Pregnancy, Type 1 diabetes mellitus, Systematic review, Meta-analysis

## Abstract

**Aims:**

To assess the efficacy and safety of automated insulin delivery (AID) systems compared to standard care in managing glycaemic control during pregnancy in women with Type 1 Diabetes Mellitus (T1DM).

**Methods:**

We searched MEDLINE, Cochrane Library, registries and conference abstracts up to June 2024 for randomized controlled trials (RCTs) and observational studies comparing AID to standard care in pregnant women with T1DM. We conducted random effects meta-analyses for % of 24-h time in range of 63–140 mg/dL (TIR), time in hyperglycaemia (> 140 mg/dl and > 180 mg/dL), hypoglycaemia (< 63 mg/dl and < 54 mg/dL), total insulin dose (units/kg/day), glycemic variability (%), changes in HbA1c (%), maternal and fetal outcomes.

**Results:**

Thirteen studies (450 participants) were included. AID significantly increased TIR (Mean difference, MD 7.01%, 95% CI 3.72–10.30) and reduced time in hyperglycaemia > 140 mg/dL and > 180 mg/dL (MD – 5.09%, 95% CI – 9.41 to – 0.78 and MD – 2.44%, 95% CI – 4.69 to – 0.20, respectively). Additionally, glycaemic variability was significantly reduced (MD – 1.66%, 95% CI – 2.73 to – 0.58). Other outcomes did not differ significantly.

**Conclusion:**

AID systems effectively improve glycaemic control during pregnancy in women with T1DM by increasing TIR and reducing hyperglycaemia without any observed adverse short-term effects on maternal and fetal outcomes.

**Supplementary Information:**

The online version contains supplementary material available at 10.1007/s00592-025-02446-x.

## Introduction

Pregnancy in women with Type 1 diabetes mellitus (T1DM) presents a complex clinical scenario characterized by the need for meticulous glycaemic control to ensure optimal maternal and fetal outcomes [1]. During pregnancy, the body undergoes significant adaptations in glucose regulation to ensure an adequate supply for the growing fetus, often leading to increased insulin resistance in maternal tissues [2]. This shift helps maintain the necessary glucose gradient between mother and fetus, supporting optimal fetal development. As pregnancy progresses, maternal insulin resistance increases, prioritizing glucose for fetal use while maternal tissues rely more on alternative energy sources. This adaptation is crucial to avoid excessive glucose exposure to the fetus, which could impact its growth and health [2]. Historically, achieving tight glycaemic control during pregnancy has been challenging, with the risk of maternal hyperglycaemia leading to adverse pregnancy outcomes such as macrosomia, preterm birth, and neonatal hypoglycaemia [3]. Conventional insulin therapy regimens, including multiple daily injections (MDI) and continuous subcutaneous insulin infusion (CSII), have been the mainstay of management in this population [4]. However, these approaches often fall short in replicating the intricate dynamics of endogenous insulin secretion, particularly in response to prandial glucose excursions [5].

In recent years, advancements in diabetes technology have introduced the era of closed-loop systems, offering the promise of enhanced glycaemic control through the automation of insulin delivery. Closed-loop systems, also known as artificial pancreas systems, utilize continuous glucose monitoring (CGM) data to inform insulin delivery, thereby mimicking aspects of physiological insulin secretion more closely than conventional therapy modalities [6]. Within this landscape, Automated insulin delivery (AID) systems have emerged as notable innovations, with the potential to revolutionize diabetes management, particularly in populations such as pregnant women with T1DM [7, 8]. In a multicenter prospective cohort study comparing continuous subcutaneous insulin infusion (CSII) to multiple daily injections (MDI) in women with pregestational type 1 diabetes mellitus (T1DM), CSII users achieved better glycaemic control in the third trimester, showing a lower HbA1c and a higher rate of HbA1c within target range compared to those on MDI [9] Real-world data further support the efficacy of AID systems, with a substantial proportion of users achieving glycaemic targets and improved time-in-range metrics [10].

Complications associated with pregnancies in women with T1DM, including congenital anomalies, miscarriages, preeclampsia, preterm delivery and large for gestational age (LGA) neonates, underscore the critical need for effective management strategies [11]. While both MDI and insulin pumps have demonstrated efficacy in T1DM management during pregnancy, AID systems offer additional benefits, adjusting basal rate to glucose values derived from CGM and in some systems providing additional boluses for correction of increased glucose levels [12]. Although most commercially available AID systems are not currently approved for pregnancy use, their potential to improve glycaemic control and pregnancy outcomes warrants further investigation. This systematic review and meta-analysis aim to assess the efficacy and safety of AID systems in pregnant women with T1DM.

## Materials and methods

This systematic review and meta-analysis adhered to the guidelines outlined in the Preferred Reporting Items for Systematic Reviews and Meta-Analyses (PRISMA) guidelines. The protocol has been registered in the PROSPERO database (registration number CRD42024578637).

### Data sources

A comprehensive search was conducted in MEDLINE (via PubMed) and the Cochrane Central Register of Controlled Trials (CENTRAL) day. Additional searches were performed manually in ClinicalTrials.gov and other trial registries to ensure all relevant studies were identified. American Diabetes Association (ADA), European Association for the Study of Diabetes (EASD) meeting, Advanced Technologies & Treatments for Diabetes (ATTD) and European Society for Paediatric Endocrinology (ESPE) annual meetings were also searched for relative conference abstracts. In cases where articles were not readily accessible through databases or institutional subscriptions, the authors were contacted to request copies of their publications. The search strategy included medical subject heading (MeSH) terms and free-text terms such as "Automated Insulin Delivery", "Closed-Loop", "Type 1 Diabetes Mellitus" and "Pregnancy". The full search strategy is detailed in Supplementary (Table S1).

### Study selection

We included randomized controlled trials (RCTs) and observational studies that evaluated the impact of AID systems on glycaemic control in pregnant women with T1DM. Controls included pregnant woman with T1DM on sensor-augmented pump or multiple daily insulin injections. Only studies published in English were considered. Studies involving other forms of diabetes or non-pregnant patients were excluded. Case studies or case series (≤ 5 cases) were also excluded. Search results were imported into reference management software for deduplication. Two independent reviewers (A.S and A.C.) screened the titles and abstracts of all records. Full-text articles of potentially eligible studies were then reviewed against pre-specified inclusion criteria: (1) RCTs and observational studies; (2) enrolling > 5 pregnant women with T1DM; (3) comparing AID systems to sensor-augmented pump or multiple daily insulin injections; and (4) those reporting any of the outcomes of interest. Any disagreements were resolved by discussion until the reviewers reached consensus.

### Data extraction

Data extraction was carried out using pre-designed extraction forms in Microsoft Excel^®^ software program for Windows (Microsoft Corporation, Redmond, WA, USA). Extracted data included study characteristics, participant demographics, intervention details and outcome data. The primary outcome was the percentage of time spent in the target glucose range (TIR) of 63–140 mg/dL. Secondary outcomes included time below 63 mg/dL, time below 54 mg/dL, time above 140 mg/dL, time above 180 mg/dL, changes in glycated hemoglobin (HbA1c), total daily insulin dose (units/kg/day) and glycaemic variability (CV, %). Safety outcome included number of episodes of severe hypoglycaemia (patients experiencing at least one hypoglycaemic event requiring assistance). TIR 63–140 mg/dl, time below 63 mg/dl and above 140 mg/dl, changes in HbA1c and glycaemic variability (CV, %) were evaluated separately for each pregnancy trimester when such data were available. Additionally, maternal outcomes included gestational weight gain (kg), percentage of cesarean delivery and incidence of preeclampsia. Fetal health outcomes included birth weight (g), incidence of macrosomia neonates born > 4 kg, incidence of neonates born LGA and small for gestational age (SGA), proportion of neonates who were admitted to NICU > 24 h and duration of hospitalization, incidence of preterm deliveries, incidence of hypoglycaemia episodes requiring glucose and incidence of still-neonatal deaths (< 7 days) were recorded. Two independent reviewers (A.S. and A.C.) conducted the data extraction, and any discrepancies were resolved by consensus. If outcome data were solely available in graphical form, we employed WebPlotDigitizer Version 5.4 (Automeris LLS, Pacifica, CA, USA) to convert them into numerical values [13]. For single-arm observational studies comparisons were made either with the initial visit, the pregestational phase, the run-in period, or early pregnancy, depending on the comparator used in each respective study. When a study did not report standard deviation (SD), standard error (SE) or 95% confidence interval (CI), we calculated the standard deviation using the p-value when available [14]. If this approach was not feasible, we used the standard deviation from a comparable study with a similar participant number [15]. If data were presented separately for each trimester of pregnancy, the average values across the three trimesters were calculated. In cases where glycaemic variability was reported as the SD of mean glucose, the CV was calculated by dividing the SD with the mean glucose level of each study. CV < 36% was considered optimal [16]. Finally, if total insulin dose was reported in units per day, the dose per kilogram (units/kg/day) was calculated by dividing it with the average weight (kg) of participants in each study, where available.

### Risk of bias assessment

Two reviewers (A.S. and A.C.) independently assessed the risk of bias for each RCT for the primary outcome (TIR) using the revised Cochrane Collaboration Risk of Bias tool (RoB 2.0) [17] and each observational study using the Risk of Bias In Non-randomized Studies of Interventions (ROBINS-I) tool [17, 18]. Each domain was evaluated and studies were categorized as having low, moderate, or high risk of bias. The overall risk of bias was categorized as low if all domains were rated as low risk, high if at least one domain was rated as high risk, and as having some concerns in all other cases. Any discrepancies were resolved by discussion until an agreement was reached.

### Data synthesis

Statistical analyses were performed using R-Studio software (version 4.2.0) and the R package ‘meta’ (R Foundation for Statistical Computing, Vienna, Austria). Meta-analyses were conducted for outcomes reported in at least two studies. For continuous outcomes, mean differences (MDs) with 95% confidence intervals (CIs) were calculated using an inverse variance random effects model. For dichotomous outcomes, odds ratios (ORs) with 95% CIs were calculated using a random effects Mantel–Haenszel model. Heterogeneity among studies was assessed using the I^2^ statistic, with values greater than 60% indicating substantial heterogeneity [15]. A *P* value for subgroup differences (p-interaction) ≤ 0.05 was considered statistically significant. Additionally, sensitivity analyses were conducted for the same outcomes based on the risk of bias, excluding studies with some concerns or high risk.

### Certainty of evidence

The Grading of Recommendations, Assessment, Development, and Evaluation (GRADE) methodology [19] was employed to evaluate the certainty of the evidence regarding the outcomes of TIR, changes in HbA1c and glycaemic variability. GRADE assessment was conducted separately for RCTs and observational studies. The evidence was deemed to be of high certainty if there was strong confidence that the true effect was close to the estimated effect. It was considered of moderate certainty if the true effect was likely to be close to the estimate, low certainty if confidence in the estimate was limited, and very low certainty if there was very little confidence in the estimate.

## Results

### Search results and study characteristics

**Fig. 1:**
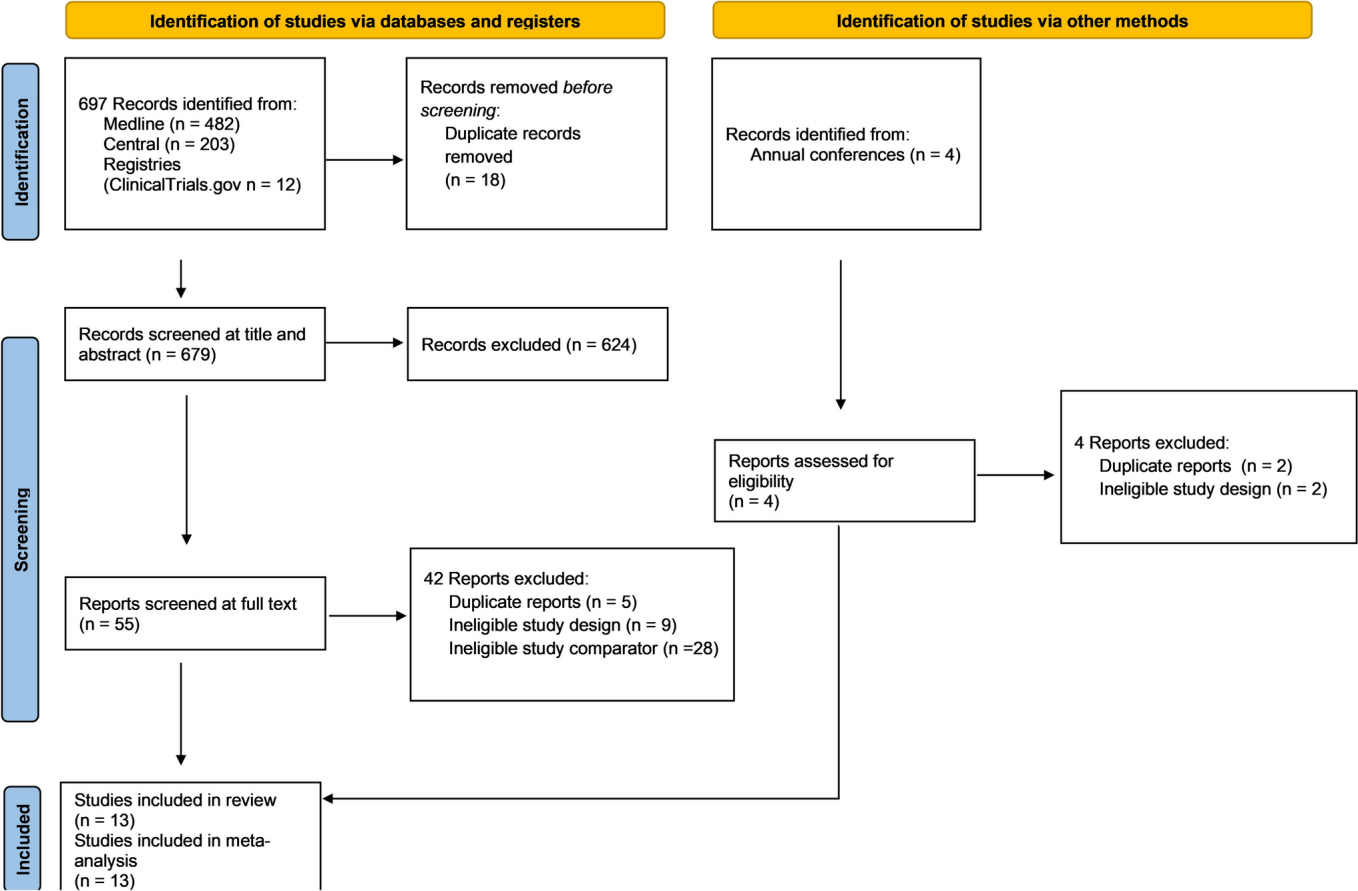
Preferred Reporting Items for Systematic reviews and Meta-Analyses (PRISMA) flow chart for the identification inclusion and exclusion of studies

The flow diagram of the study selection process is presented in Fig. 1. A total of 697 records were identified through a comprehensive search, including records from databases (Medline, Central, ClinicalTrials.gov) and annual conferences. After removing 18 duplicate records, 679 records were screened based on titles and abstracts. Of these, 624 records were excluded as they did not meet the inclusion criteria. Full-text reviews were conducted for 55 reports, of which 42 were excluded due to reasons such as duplicate reports, ineligible study designs, and inappropriate comparators. 13 studies were included in this systematic review and meta-analysis, comprising a total of 450 participants. Among the included studies, six were RCTs [20–25] and seven were observational studies [26–32]. Of the RCTs, three had crossover design [20, 24, 25]. Of the seven observational studies included, two were cohort studies [31, 32]. Baseline characteristics of the study populations are summarized in Table 1. Risk of bias assessment for RCTs was deemed low overall, aside from one study which was deemed to be of some concerns [23], while risk of bias assessment for observational studies was low overall, except from three studies, two of which were of high risk [28, 31] and one was of some concerns [26].

**Table 1:** Study-level and participant baseline characteristics included in the meta-analysis

Study	Study design	Patients randomized, n	Mean age, years	Mean BMI kg/m^2^	Type of AID systems	Duration of T1DM, years	Mean HbA1c, %	Total daily insulin dose, U/kg	Duration of gestation, weeks ± SD	Duration of intervention, weeks
Murphy 2011ISRCTN50385583 [20]	Randomized crossover	12	33.22 ± 1.92	76.77 ± 4.92	FreeStyle Navigator, J&J Animas 2020	17.6 ± 5.9	6.37 ± 0.15	NA	19.75 ± 2.75	2 24 h (19th and 23rd gestational week)
Stewart 2016 ISRCTN71510001 [25]	Randomized crossover	16	34.1 ± 4.6	29.7 ± 5.7	FreeStyle Navigator II, DANA Diabecare R	23.6 ± 7.2	6.8 ± 0.6	52.8 ± 18.1 (U)	14 ± 3.3	4 weeks
Stewart 2018 ISRCTN83316328 [24]	Randomized crossover	16	32.8 ± 5	26.6 ± 4.4	FreeStyle Navigator II, DANA Diabecare R	19.4 ± 10.2	8 ± 1.1	0.51 ± 0.09	16.4 ± 4.9	4 weeks
Benhalima 2024 NCT04520971 [21]	Randomized parallel	95	30.5 ± 4.2	26.5 ± 4.6	Medtronic Guardian 3 + 4, MiniMed 780G	16.8 ± 8	6.5 ± 0.6	0.6 ± 0.2	10.1 ± 0.6	10th gestational week until delivery
Lee 2023 ISRCTN56898625 [23]	Randomized parallel	124	31.1 ± 5.3	27.4 ± 5.35	Dexcom G6—Dana Diabecare RS	17 ± 7.5	7.7 ± 1.2	0.7 ± 0.2	11.15 ± 0.66	9.6th gestational week until 6 months postpartum
Polsky 2024 NCT03774186 [22]	Randomized parallel	24	31.1 ± 4.2		Medtronic Guardian 3 -MiniMed 670G	19.3 ± 7.65	6.8 ± 0.85	29 ± 11.25 (u/d)	8.5 ± 1.6	< 11th gestational week until 6 weeks postpartum
Albert 2023 [26]	real-life retrospective single-group study	6	33.25 ± 3.12	26.75 ± 3.97	Minimed 780G	20.5 ± 3.12	6.55 ± 0.15	NA	NA	4th gestational week until delivery
Dodesini 2023 [27]	retrospective single-center real-life	8	32.4 ± 4.5	23.7 ± 3.3	Minimed 780G	19.6 ± 4.5	6.8 ± 0.8	NA	NA	13th to 36th gestational week
Guibert 2023 [28]	observational multicenter retrospective study	13	33.1 ± 3.2	26.9 ± 3.8	MiniMed 780G	19.3 ± 6.9	7.3 ± 0.7	0.72 ± 0.21	NA	38 weeks
Levy 2023 [29]	single-arm observational multicenter study	10	32.6 ± 4.3	27.2 ± 4.9	Tandem t: AP, a continuous glucose monitor, and a MPC–based algorithm customized for pregnancy (CLC-P)	16.6 ± 7.8	5.8 ± 0.6	0.55 ± 0.18	22 ± 3.4	14th gestational week until delivery
Fresa 2024 [30]	single-arm observational study	6	34 ± 3.51	21.97 ± 2.37	MiniMed 780G	15.75 ± 9.75	6.45 ± 0.31	0.48 ± 0.16	NA	Pregestational phase (14 days before the first day of the last menstrual period) to the first 30 days postpartum
Nandam 2024 [31]	retrospective observational cohort study	8	30.6 ± 6.1	27.6 ± 6.2	Tandem t: slim X2	12.5 ± 5.8	6.7 ± 1	0.74 ± 1.72	9.3 ± 4.1	5th gestational week until delivery
Quirós 2024 [32]	Multicenter prospective cohort study	112	34.8 ± 5	25.2 ± 1.14	MiniMed 780G, Tandem Control IQ, and Diabeloop	17 ± 8.9	6.7 ± 0.15	NA	8.02 ± 0.88	6th gestational week until 4 weeks after birth

### Time in ranges

**Fig. 2:**
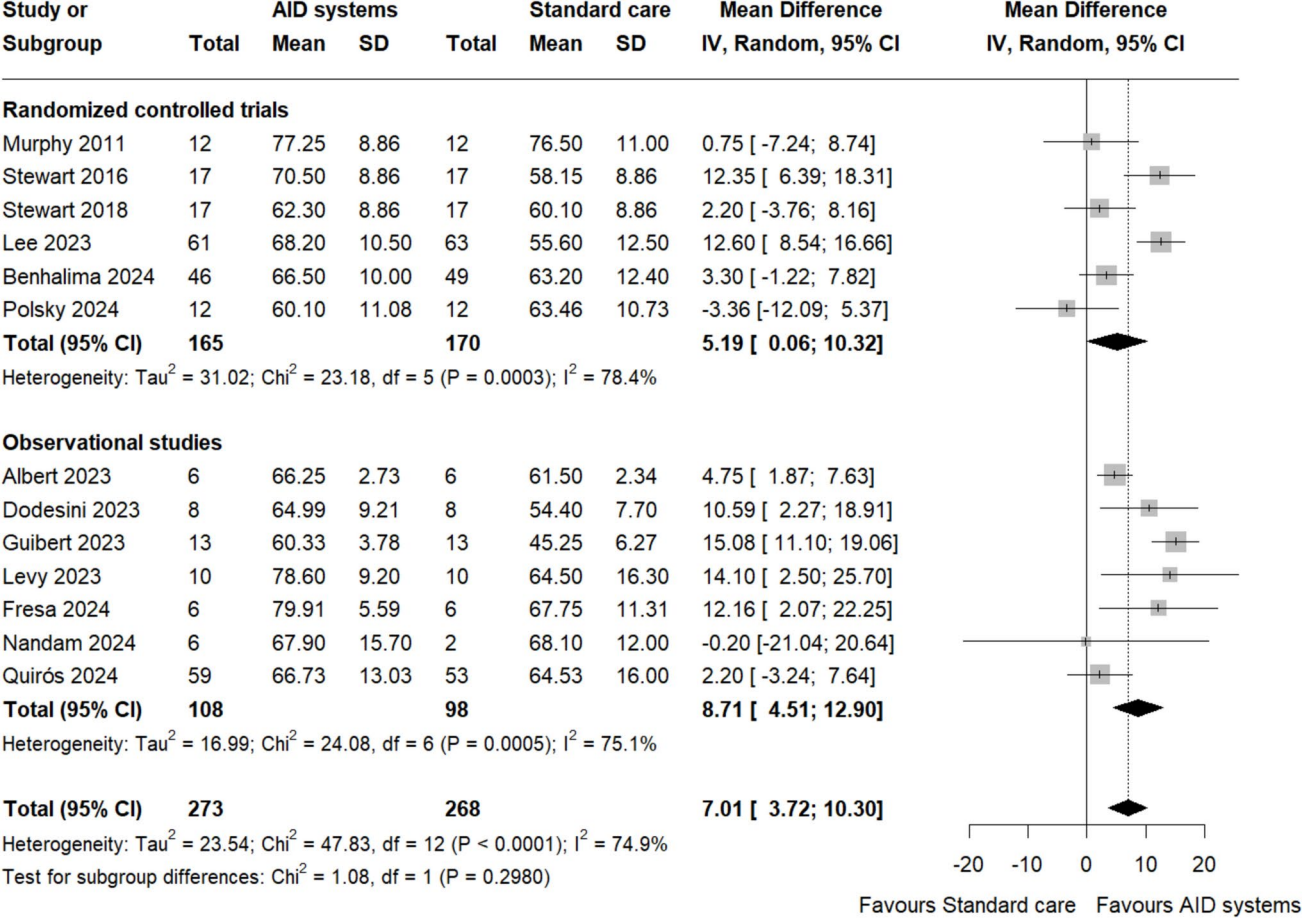
Forest plot of time in range (TIR, 24-h percentage) 63–140 mg/dl. Overall effect and subgroup effects based on study design

**Fig. 3:**
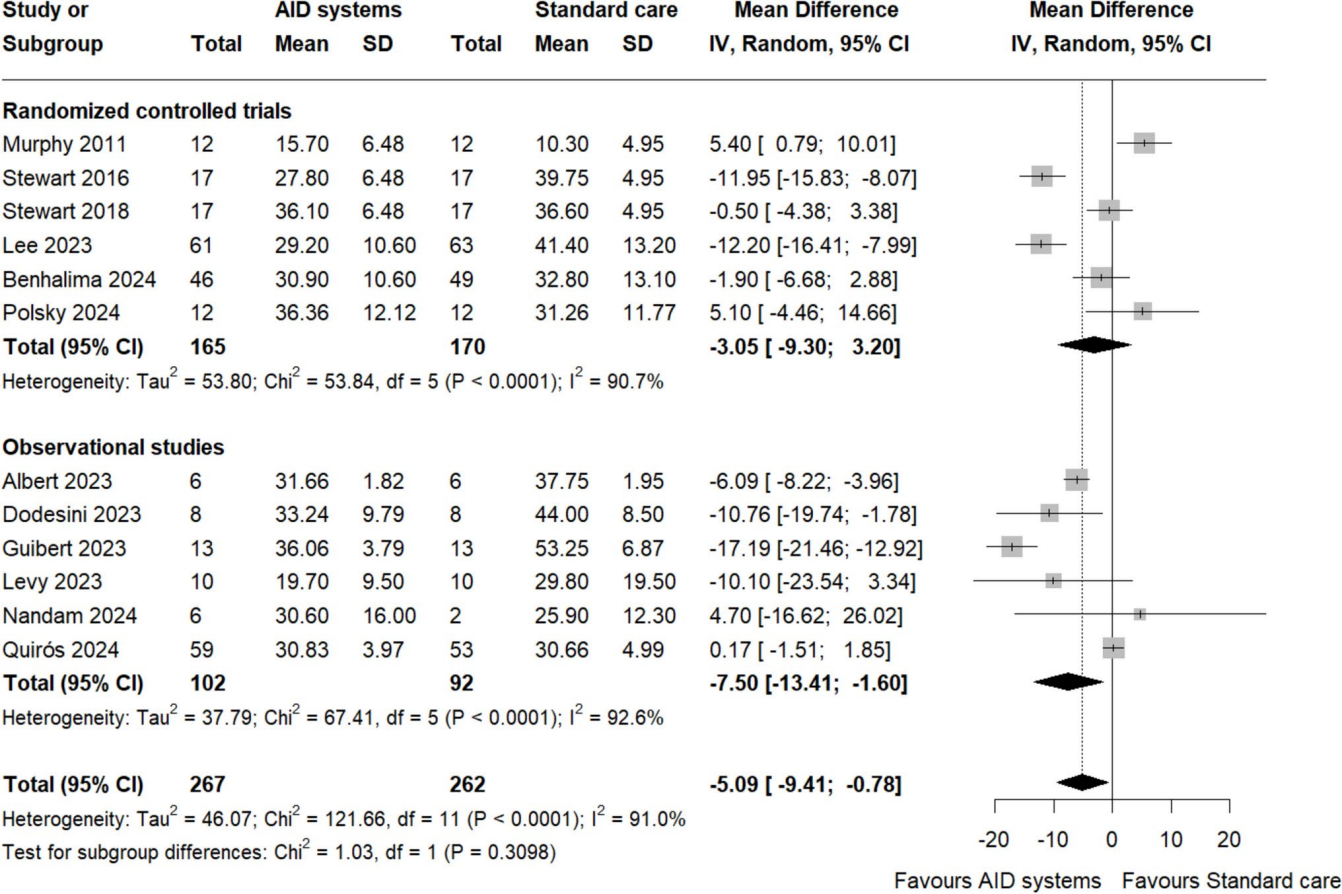
Forest plot of time spent in hyperglycaemia > 140 mg/dl. Overall effect and subgroup effects based on study design

13 studies provided data for TIR [20–32]. The use of AID systems in pregnant women with T1DM resulted in a higher percentage of TIR compared with standard care (MD 7.01%, 95% CI 3.72–10.30, I^2^ = 74.9%, Fig. 2). The level of certainty for this finding was moderate for RCTs (Table S1) and low for observational studies (Table S2). Time in hypoglycaemia < 63 mg/dL and < 54 mg/dl did not differ among AID systems and standard care (MD – 0.25%, 95% CI – 1.22 to 0.72, I^2^ = 95.3% and MD – 0.35%, 95% CI – 0.70 to 0.00, I^2^ = 72.9%, respectively, Figure S3 and S4, respectively). Patients using AID systems spent less time in hyperglycaemia > 140 mg/dL and > 180 mg/d compared to patients using standard care (MD – 5.09%, 95% CI – 9.41 to – 0.78, I^2^ = 91% and MD – 2.44%, 95% CI – 4.69 to – 0.20, I^2^ = 90.4%, Fig. 3 and S5, respectively).

Subgroup analyses for TIR suggested that the beneficial effect of AID systems occurred irrespective of study type, as no statistically significant subgroup difference was found (P for subgroup differences > 0.05, Fig. 2). Similarly, no significant subgroup effects were evident in the analyses for time spent in hypoglycaemia < 63 mg/dL or hyperglycaemia > 140 mg/dL and > 180 mg/dL (Fig. 3 and S5, respectively). Subgroup analysis for time spent < 54 mg/dl indicated a statistically significant difference between the subgroups (P for subgroup differences = 0.0010), with the observational subgroup showing less time in hypoglycaemia compared to RCTs. Sensitivity analyses indicated that the results for TIR remained statistically significant (MD 5.46%, 95% CI 1.76–9.15, I^2^ = 57.7%, Figure S6) and time spent in hypoglycaemia < 54 mg/dL became statistically significant (MD – 0.72%, 95% CI – 1.09 to – 0.36, I^2^ = 0%, Figure S7), indicating that the findings were strengthened after excluding studies with moderate or high risk of bias. Sensitivity analysis for time spent < 63 mg/dl and time spent in hyperglycaemia > 140 mg/dl and > 180 mg/dl showed no significant differences.

### Glycaemic variability and HbA1c

**Fig. 4:**
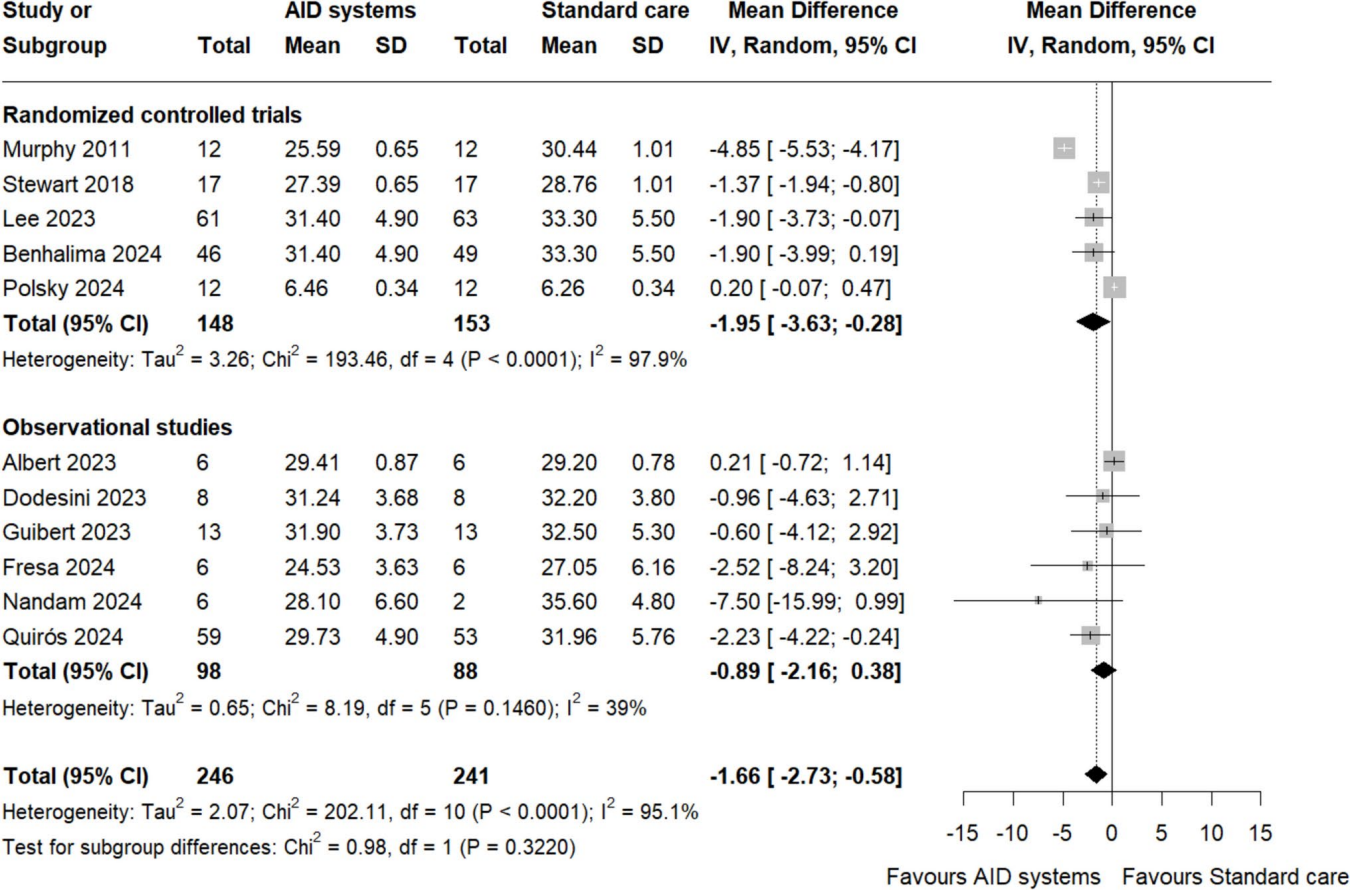
Forest plot of glycaemic variability (CV, %). Overall effect and subgroup effects based on study design

A total of 11 studies with 246 participants in the AID system group and 241 participants in the standard care group provided data for glycaemic variability [20–24, 26–28, 30–32]. The pooled analysis demonstrated a statistically significant reduction in glycaemic variability for AID systems compared to standard care, (MD – 1.66%, 95% CI – 2.73 to – 0.58, I^2^ = 95.1%, Fig. 4). The level of certainty for this finding was high for RCTs and very low for observational studies (Table S2). For HbA1c, a total of 9 studies with 226 participants in the AID system group and 221 participants in the standard care group were included [21–23, 25, 26, 28, 30–32]. The analysis showed no significant difference between AID systems and standard care (MD – 0.15%, 95% CI – 0.40 to 0.09, I^2^ = 78.6%, Figure S8). The level of certainty for this finding was low for RCTs and very low for observational studies (Table S2).

Based on subgroup analysis there were no statistically significant differences between the RCTs and observational studies for both glycaemic variability (P-value = 0.32, Fig. 4) and HbA1c (P-value = 0.19, Figure S8). Sensitivity analysis indicated that the results for glycaemic variability remained significant (MD – 1.94%, 95% CI – 3.44 to – 0.44, I^2^ = 96.9% Figure S9), indicating a consistent benefit of AID systems over standard care. Similarly, for HbA1c sensitivity analysis did not alter the overall findings, with the estimates remaining non-significant (MD 0.00%, 95% CI – 0.32 to 0.32, I^2^ = 78.1%, Figure S10).

### Total daily insulin

Total daily insulin dose did not differ between the AID systems and standard care groups (MD 0.09 units/kg/day, 95% CI – 0.00 to 0.19, I^2^ = 42.9%, Figure S11). Subgroup analysis indicated a statistically significant difference between RCTs and observational studies (P for subgroup differences = 0.03, Figure S11), showing a greater effect of AID systems on increasing total insulin dose compared to RCTs. Sensitivity analysis did not change the overall findings (MD 0.12 units/kg/day, 95% CI 0.02–0.42, I^2^ = 52.9%, Figure S12).

### Severe hypoglycaemia

Total number of events of severe hypoglycaemia showed no statistically significant difference between the AID system and standard care groups (OR 1.02, 95% CI 0.39–2.66, I^2^ = 32.1%, Figure S13). Similarly, no subgroup effect was evident (P for subgroup analysis = 0.30, Figure S13). Additionally, sensitivity analysis did not differ after exclusion of some concerns and high risk of bias studies.

### Maternal postpartum outcomes

According to maternal outcomes, the use of AID systems did not result in significant weight gain during pregnancy (MD – 0.96 kg, 95% CI – 3.94 to 2.01, I^2^ = 87.5%, Figure S14), and did not show a statistically significant difference compared to standard care in incidence of preeclampsia (OR 0.74, 95% CI 0.30–1.85, I^2^ = 35%, Figure S15) or percentage of cesarean sections performed (OR 0.90, 95% CI 0.50–1.63, I^2^ = 31.7%, Figure S16). Based on subgroup analyses, RCTs indicated that participants in the AID system group had significantly less weight gain during pregnancy compared to those receiving standard care (MD – 2.53 kg, CI – 3.91 to – 1.15, I^2^ = 0%, P for subgroup differences < 0.01). In contrast, the observational study suggested the opposite (Figure S14). Subgroup analysis for incidence of preeclampsia and percentage of cesarean sections performed suggests that study design did not affect the results. Sensitivity analyses for all three outcomes did not show significant difference between the two groups.

### Fetal outcomes

Based on the analysis of fetal outcomes, including birth weight (g), incidence of neonates born with macrosomia (> 4 kg), incidence of LGA and SGA neonates, proportion and duration of NICU admissions (> 24 h), incidence of preterm deliveries, incidence of hypoglycaemia events requiring glucose, and incidence of stillbirths or neonatal deaths (< 7 days), no statistically significant differences were observed between the AID system and standard care groups (Figures S17–S25). Subgroup analyses indicated that results were not affected by study design for all outcomes, aside from the incidence of LGA neonates where, RCTs suggested a trend towards a lower risk of LGA neonates in the AID system group, whereas observational studies tended to show the opposite effect (Figure S19). Neither of the effects were statistically significant. Subgroup analyses indicated that results were not affected by study design for all outcomes. Sensitivity analyses for all outcomes indicated no significant difference between AID systems and standard care.

### Trimesters

The first trimester analysis revealed that the use of AID systems significantly increased the time spent within the target glucose range of 63–140 mg/dl compared to standard care (MD 5.79%, 95% CI 3.41–8.16, I^2^ = 27.1%, Figure S26) and significantly reduced the time spent above 140 mg/dl (MD – 5.21%, 95% CI – 9.78 to – 0.63, I^2^ = 85%, Figure S27). There was no significant difference in the time spent below 63 mg/dl (MD 0.35%, 95% CI – 0.78 to 1.48, I^2^ = 97.5%, Figure S28), changes in HbA1c levels (MD – 0.17%, 95% CI – 0.34 to – 0.01, I^2^ = 6.1%, Figure S29), or glycaemic variability as measured by CV% (MD – 1.30%, 95% CI – 5.20 to 2.61, I^2^ = 66.4%, Figure S30). During the second trimester, analyses revealed that TIR 63–140 mg/dl was significantly higher in those using AID systems compared to standard care (MD 6.37%, 95% CI 1.33–11.42, I^2^ = 77.7%, Figure S31). There was no significant difference in time spent below 63 mg/dl (MD – 0.40%, 95% CI – 2.60 to 1.81, I^2^ = 97.6%, Figure S32), time above 140 mg/dl (MD – 4.61%, 95% CI – 11.90 to 2.69, I^2^ = 91.7%, Figure S33), changes in HbA1c (MD – 0.27%, 95% CI – 0.66 to 0.12, I^2^ = 86%, Figure S34) and glycaemic variability (MD – 1.02%, 95% CI – 2.86 to 0.83, I^2^ = 69.9%, Figure S35). For the third trimester, patients using AID systems had lower glycaemic variability compared to standard care (MD – 1.66%, 95% CI – 2.29 to – 1.03, I^2^ = 0%, Figure S36). There was no significant difference between AID systems and standard care in TIR 63–140 mg/dl (MD 7.12%, 95% CI – 0.45 to 14.69, I^2^ = 90.4%, Figure S37), time below 63 mg/dl (MD – 1.39%, 95% CI – 3.53 to 0.75, I^2^ = 98.5%, Figure S38), time above 140 mg/dl (MD – 4.59%, 95% CI – 14.75 to 5.56, I^2^ = 96.9%, Figure S39), or HbA1c changes (MD – 0.18%, 95% CI – 0.64 to 0.28, I^2^ = 86.1%, Figure S40).

## Discussion

In this systematic review and meta-analysis, we aimed to assess the efficacy and safety of AID systems in pregnant women with T1DM. The findings suggest that the use of AID systems was consistently associated with an improvement in glycaemic control throughout pregnancy. Specifically, the use of AID systems was associated with a notable increase in TIR across all three trimesters. Furthermore, AID systems effectively reduced the time spent in hyperglycaemia during the first trimester and significantly lowered glycaemic variability mainly during the third trimester. No significant differences were observed in maternal or fetal outcomes between the use of AID systems and standard care.

Previous meta-analyses have also evaluated the efficacy of AID systems in pregnant women with T1DM [33, 34]. Lei et al. (2024) conducted a meta-analysis involving four RCTs with a total of 164 participants [33]. Their findings showed a significant improvement in 24-h TIR and nocturnal TIR with AID systems compared to standard care, particularly emphasizing the overnight period. No significant differences were observed in the 24-h time below or above range and the study was limited by its small sample size and the exclusion of observational studies [33]. Our analysis included two more RCTs and seven observational studies involving a total of 450 patients. Our results demonstrated a consistent improvement in TIR across all trimesters and a significant reduction in glycaemic variability. More recently, Teixeira et al. (2024) analyzed data from five RCTs involving 236 pregnant women [34]. While they found no significant difference in overall TIR between AID systems and standard care, they did observe improvements in nocturnal TIR and glucose variability. However, their analysis was limited by the smaller number of included studies and did not fully explore the potential impact of AID systems across different trimesters or in observational settings. Our study included one additional RCT and observational data, which provided a more robust and conclusive analysis. Our findings also encompass a broader range of variables, including glycaemic variability, maternal and fetal outcomes. Furthermore, to enhance the clinical interpretation of our findings, we conducted subgroup and sensitivity analyses to address the heterogeneity introduced by different AID systems and algorithms and to ensure the robustness of our results.

Our meta-analysis demonstrated that AID systems use during pregnancy in women with T1DM significantly increased time spent in normoglycaemia by 7.01%. This increase corresponds to an additional 100 min approximately per day that pregnant women spent within the target glucose range. The beneficial effect of AID systems was consistent throughout pregnancy, with TIR increasing by 83.3 min per day in the first trimester, 91.73 min/day in the second trimester, and 102.53 min/day in the third trimester. Although the increase in the third trimester was not statistically significant, it remains clinically meaningful. In align with our findings, a systematic review of 59 studies evaluating various hybrid closed-loop (HCL) systems, including real-world and clinical trial data, also confirmed the effectiveness of these systems in improving TIR in patients with T1DM [12], while a study evaluating the Medtronic Minimed 780G HCL system reported similar progressive improvements in TIR throughout pregnancy, with TIR increasing from 64.0% in the first trimester to 75.7% in the third trimester [10]. Furthermore, the use of AID systems contributed to a reduction in time spent in hyperglycemia, decreasing it by 73.34 min per day for glucose levels > 140 mg/dL and by 35.14 min per day for levels > 180 mg/dL. This reduction can be attributed to the advanced algorithms in AID systems, which continuously monitor glucose levels and adjust insulin delivery in real-time, thereby preventing prolonged periods of hyperglycaemia and maintaining tighter glycaemic control [35, 36]. Despite these improvements, it should be noted that TIR values achieved with AID systems in most studies remain below the recommended target of 70%, with TAR values often exceeding 25%, potentially impacting fetal outcomes, which were not significantly different from those in standard care groups. This suggests that while AID systems enhance glycaemic control, its clinical impact on pregnancy outcomes may be limited. No significant differences were observed in time spent in hypoglycaemia, although subgroup analysis revealed that RCTs showed significantly less time in hypoglycaemia < 54 mg/dl potentially due to real-world settings [37]. Despite the known increased risk of hypoglycaemia during the first and early second trimesters [38], our findings revealed no significant differences between the two systems in terms of hypoglycaemia duration across trimesters.

Moreover, our analysis demonstrated a significant reduction in glycaemic variability, particularly during the third trimester, which is crucial as lower glycaemic variability facilitates achieving tighter glucose control targets and reduces the risk of neonatal hypoglycaemia [39]. However, we found no significant difference in HbA1c levels between AID systems and standard care. Achieving the recommended HbA1c targets (≤ 6.5% in early pregnancy and < 6.0% during pregnancy) remains highly challenging, as evidenced by previous studies showing that only a minority of pregnant women with T1DM successfully reach these goals [40–42]. Additionally, there was no significant difference in total insulin dose between the AID systems and standard care. However, subgroup analysis revealed that AID systems had a greater impact on increasing insulin doses in observational studies compared to RCTs, reflecting the real-world adjustments made to accommodate the doubling of insulin requirements typically observed during pregnancy [43, 44]. There was also no significant difference in the total number of severe hypoglycaemia events between AHCLS and standard care, although most studies reported zero episodes. However, it is well-documented that pregnant women with T1DM face a particularly high risk of severe hypoglycaemia, especially during the first and second trimesters, underscoring the need for vigilant monitoring [45, 46].

The use of AID systems did not significantly differ from standard care regarding maternal outcomes, including weight gain during pregnancy, preeclampsia and cesarean sections. Interestingly, while RCTs indicated that AID systems was associated with significantly less weight gain compared to standard care, the observational study suggested the opposite, highlighting the discrepancy in results across different study designs. Literature confirms that pregnant women with T1DM have an increased risk of adverse outcomes, including preeclampsia and cesarean delivery, largely driven by maternal hyperglycaemia [3, 11]. The ability of AID systems to reduce time spent in hyperglycaemia may contribute to more favorable weight management outcomes during pregnancy. Additionally, excessive gestational weight gain, particularly in those with higher preconception BMI, is associated with an increased risk of perinatal complications [47]. Finally, our analysis of fetal outcomes showed no statistically significant differences between the AID systems and standard care groups, with both treatments yielding similar results for birth weight, macrosomia, incidence of SGA and LGA neonates, NICU admissions, preterm deliveries, hypoglycaemia events requiring glycose and neonatal deaths. However, subgroup analyses highlighted discrepancy due to study design. Although not statistically significant, clinically it appears that in the RCTs, 53 out of 119 neonates in the AID system group were LGA compared to 67 out of 124 in the standard care group, while in the observational studies, 43 out of 64 neonates in the AID system group were LGA compared to 30 out of 54 in the standard care group. Studies have shown that women with T1DM are at an increased risk of delivering LGA and SGA neonates, compared to pregnancies without diabetes [48]. This emphasizes the importance of strict glycaemic control throughout pregnancy to mitigate these risks and improve perinatal outcomes.

Several limitations should be acknowledged. Although our analysis included both RCTs and observational studies, the heterogeneity across these studies was substantial, particularly in outcomes related to time in ranges, glycaemic variability, HbA1c, birth weight, weight gain and NICU days. High levels of statistical heterogeneity, especially in TIR < 63 mg/dL and < 54 mg/dL, were partly mitigated through subgroup analyses focused on randomized trials, while sensitivity analyses improved the consistency of TIR outcomes. Nevertheless, this variability suggests underlying clinical differences among the studies in study design, duration and patient populations, which may limit the generalizability of our findings. The heterogeneity between studies is also increased by the use of different AID systems with varying algorithms and glucose targets, as well as by interventions like'fake carbs' to avoid postprandial hyperglycaemia [21]. Additionally, the inclusion of observational studies, while broadening the scope of the analysis, may import potential confounding factors that cannot be fully accounted for, underscoring the need for more high-quality, long-term RCTs. Moreover, the observational studies included, varied in their choice of control groups, with some comparing AID systems outcomes against initial visit metrics, the pregestational phase, the run-in period, or early pregnancy data, depending on the specific study design. These comparisons may not be directly comparable due to the physiological changes that occur during pregnancy, which can significantly influence glycaemic control and insulin sensitivity [49]. Furthermore, in cases where SD, SE, or 95% CIs were not reported, we approximated SD using available p-values or utilized values from comparable studies. This approach introduces potential variability and reduces the precision of the results. Finally, most of the included studies had small sample sizes, potentially limiting the statistical power to detect differences in secondary outcomes. Thus, while our findings provide valuable insights, they should be interpreted with caution, and there remains a need for more high-quality, long-term RCTs to confirm these results and to investigate t complex relationship between advanced insulin delivery technologies and the dynamic physiological adaptations of pregnancy.

In conclusion, our findings suggest that the use of AID systems in pregnant women with T1DM provides significant improvements in glycaemic control throughout pregnancy. The consistent increase in TIR across all trimesters, along with the reduction in glycaemic variability, highlights the potential of AID systems to optimize both maternal and fetal outcomes. While no significant differences were observed in maternal and fetal complications, the enhanced precision of glycaemic control offered by AID systems underscores its promise as a preferable therapeutic option. Given the limitations of the current evidence, particularly the heterogeneity among studies and the need for long-term data, further well methodologically designed RCTs are warranted to confirm these findings and add on to the existing literature on both short-term and long-term maternal and fetal benefits of AID systems throughout pregnancy.

## Supplementary Information

Below is the link to the electronic supplementary material.

Supplementary file1 (DOCX 1702 KB)

Supplementary file2 (DOCX 32 KB)

## Data Availability

The data that support the current study are available from the corresponding author upon reasonable request.
