# Supplementary material for: Automated insulin delivery in pregnant women with type 1 diabetes mellitus: a systematic review and meta-analysis
Source: Acta Diabetol. 2025 Jan 10;62(4):441–52. doi: 10.1007/s00592-025-02446-x (PMC12055664; doi:10.1007/s00592-025-02446-x)

**Automated insulin delivery in pregnant women with type 1 diabetes mellitus: A systematic review and meta-analysis**

**Authors**

Athina Stamati^1^, Athanasios Christoforidis^2^

**Author affiliations**

^1^ School of Medicine, Faculty of Health Science, Aristotle University of Thessaloniki, Thessaloniki, Greece

^2^ 1^st^ Paediatric Department, School of Medicine, Faculty of Health Sciences, Aristotle University of Thessaloniki, Ippokratio General Hospital, Thessaloniki, Greece

**Table of contents**

[**Table S1 – Search strategy** 8](#_Toc182073158)

[**Figure S1 - Risk of bias (RoB2) for Randomized controlled trials (RCTs)** 9](#_Toc182073159)

[**Figure S2 - Risk of bias (ROBINS-I) for observational studies** 10](#_Toc182073160)

[**Table S2 – Certainty of evidence** 11](#_Toc182073161)

[**Figure S3 - Forest plot of time spent in hypoglycaemia <63 mg/dl. Overall effect and subgroup analysis based on study design.** 12](#_Toc182073162)

[**Figure S4 - Forest plot of time spent in hypoglycaemia <54 mg/dl. Overall effect and subgroup analysis based on study design.** 13](#_Toc182073163)

[**Figure S5 - Forest plot of time spent in hyperglycaemia >180 mg/dl. Overall effect and subgroup effects based on study design.** 14](#_Toc182073164)

[**Figure S6 - Forest plot of time in range (TIR, 24-h percentage) 63-140 mg/dl after excluding studies with moderate or high risk of bias.** 15](#_Toc182073165)

[**Figure S8 - Forest plot of changes in HbA1c (%). Overall effect and subgroup analysis based on study design.** 17](#_Toc182073166)

[**Figure S10 - Forest plot of changes in HbA1c (%) after excluding studies with moderate or high risk of bias.** 19](#_Toc182073167)

[**Figure S11 - Forest plot of total daily insulin dose (units/day/kg). Overall effect and subgroup analysis based on study design.** 20](#_Toc182073168)

[**Figure S12 - Forest plot of total daily insulin dose (units/day/kg) after excluding studies with moderate or high risk of bias.** 21](#_Toc182073169)

[**Figure S13 - Forest plot of severe hypoglycaemia. Overall effect and subgroup analysis based on study design.** 22](#_Toc182073170)

[**Figure S14 - Forest plot of gestational weight gain (kg). Overall effect and subgroup analysis based on study design.** 23](#_Toc182073171)

[**Figure S15 - Forest plot of incidence of patients suffered from preeclampsia. Overall effect and subgroup analysis based on study design.** 24](#_Toc182073172)

[**Figure S16 - Forest plot of proportion of cesarean delivery. Overall effect and subgroup analysis based on study design.** 25](#_Toc182073173)

[**Figure S17 - Forest plot of birth weight (g). Overall effect and subgroup analysis based on study design.** 26](#_Toc182073174)

[**Figure S18 - Forest plot of incidence of neonates born with macrosomia >4gr. Overall effect and subgroup analysis based on study design.** 27](#_Toc182073175)

[**Figure S19 - Forest plot of incidence of LGA neonates. Overall effect and subgroup analysis based on study design.** 28](#_Toc182073176)

[**Figure S20 - Forest plot of incidence of SGA neonates. Overall effect and subgroup analysis based on study design.** 29](#_Toc182073177)

[**Figure S21 - Forest plot of proportion of NICY admissions (>24h). Overall effect and subgroup analysis based on study design.** 30](#_Toc182073178)

[**Figure S22 - Forest plot of duration of NICY admissions (days). Overall effect.** 31](#_Toc182073179)

[**Figure S23 - Forest plot of incidence for preterm delivery. Overall effect and subgroup analysis based on study design.** 32](#_Toc182073180)

[**Figure S24 - Forest plot of hypoglycaemia requiring glycose. Overall effect.** 33](#_Toc182073181)

[**Figure S25 - Forest plot of still or neonatal deaths. Overall effect and subgroup analysis based on study design.** 34](#_Toc182073182)

[**Figure S26 - Forest plot of time spent in range 63-140 mg/dl during the 1^st^ trimester. Overall effect.** 35](#_Toc182073183)

[**Figure S27 - Forest plot of time spent in hyperglycaemia >140 mg/dl during the 1^st^ trimester. Overall effect.** 36](#_Toc182073184)

[**Figure S28 - Forest plot of time spent in hypoglycaemia <63 mg/dl during the 1^st^ trimester. Overall effect.** 37](#_Toc182073185)

[**Figure S29 - Forest plot of changes in HbA1c (%) during the 1^st^ trimester. Overall effect.** 38](#_Toc182073186)

[**Figure S30 - Forest plot of glycaemic variability (CV, %) during the 1^st^ trimester. Overall effect.** 39](#_Toc182073187)

[**Figure S31 - Forest plot of time spent in range 63-140 mg/dl during the 2^nd^ trimester. Overall effect.** 40](#_Toc182073188)

[**Figure S32 - Forest plot of time spent in hypoglycaemia <63 mg/dl during the 2^nd^ trimester. Overall effect.** 41](#_Toc182073189)

[**Figure S33 - Forest plot of time spent in hyperglycaemia >140 mg/dl during the 2^nd^ trimester. Overall effect.** 42](#_Toc182073190)

[**Figure S34 - Forest plot of changes in HbA1c (%) during the 2^nd^ trimester. Overall effect.** 43](#_Toc182073191)

[**Figure S35 - Forest plot of glycaemic variability (CV, %) during the 2^nd^ trimester. Overall effect.** 44](#_Toc182073192)

[**Figure S36 - Forest plot of glycaemic variability (CV, %) during the 3^rd^ trimester. Overall effect.** 45](#_Toc182073193)

[**Figure S37 - Forest plot of time spent in range 63-140 mg/dl during the 3^rd^ trimester. Overall effect.** 46](#_Toc182073194)

[**Figure S38 - Forest plot of time spent in hypoglycaemia <63 mg/dl during the 3^rd^ trimester. Overall effect.** 47](#_Toc182073195)

[**Figure S39 - Forest plot of time spent in hyperglycaemia >140 mg/dl during the 3^rd^ trimester. Overall effect.** 48](#_Toc182073196)

[**Figure S40 - Forest plot of changes in HbA1c (%) during the 3^rd^ trimester. Overall effect.** 49](#_Toc182073197)

# **Table S1 – Search strategy**

| ("diabetes mellitus, type 1"[MeSH Terms] OR "type 1 diabetes mellitus"[All Fields] OR ("diabetes mellitus, type 1"[MeSH Terms] OR "type 1 diabetes mellitus"[All Fields] OR ("insulin"[All Fields] AND "dependent"[All Fields] AND "diabetes"[All Fields]) OR "insulin dependent diabetes"[All Fields])) AND ("pregnancy"[MeSH Terms] OR "pregnancy"[All Fields] OR "pregnancies"[All Fields] OR "pregnancy s"[All Fields] OR ("gestate"[All Fields] OR "gestated"[All Fields] OR "gestates"[All Fields] OR "gestating"[All Fields] OR "gestational"[All Fields] OR "gestations"[All Fields] OR "pregnancy"[MeSH Terms] OR "pregnancy"[All Fields] OR "gestation"[All Fields])) AND ("pump"[All Fields] OR (("automate"[All Fields] OR "automated"[All Fields] OR "automates"[All Fields] OR "automating"[All Fields] OR "automation"[MeSH Terms] OR "automation"[All Fields] OR "automations"[All Fields] OR "automation s"[All Fields]) AND ("insulin"[MeSH Terms] OR "insulin"[All Fields] OR "insulin s"[All Fields] OR "insuline"[All Fields] OR "insulinic"[All Fields] OR "insulinization"[All Fields] OR "insulinized"[All Fields] OR "insulins"[MeSH Terms] OR "insulins"[All Fields]) AND ("deliveries"[All Fields] OR "delivery, obstetric"[MeSH Terms] OR ("delivery"[All Fields] AND "obstetric"[All Fields]) OR "obstetric delivery"[All Fields] OR "delivery"[All Fields])) OR (("insulin"[MeSH Terms] OR "insulin"[All Fields] OR "insulin s"[All Fields] OR "insuline"[All Fields] OR "insulinic"[All Fields] OR "insulinization"[All Fields] OR "insulinized"[All Fields] OR "insulins"[MeSH Terms] OR "insulins"[All Fields]) AND ("infusate"[All Fields] OR "infusates"[All Fields] OR "infuse"[All Fields] OR "infused"[All Fields] OR "infuser"[All Fields] OR "infusers"[All Fields] OR "infuses"[All Fields] OR "infusing"[All Fields] OR "infusion"[All Fields] OR "infusions"[All Fields])) OR "closed-loop"[All Fields]) |
| --- |

# **Figure S1 - Risk of bias (RoB2) for Randomized controlled trials (RCTs)**

Time in range (TIR) 63-140 mg/dl

**
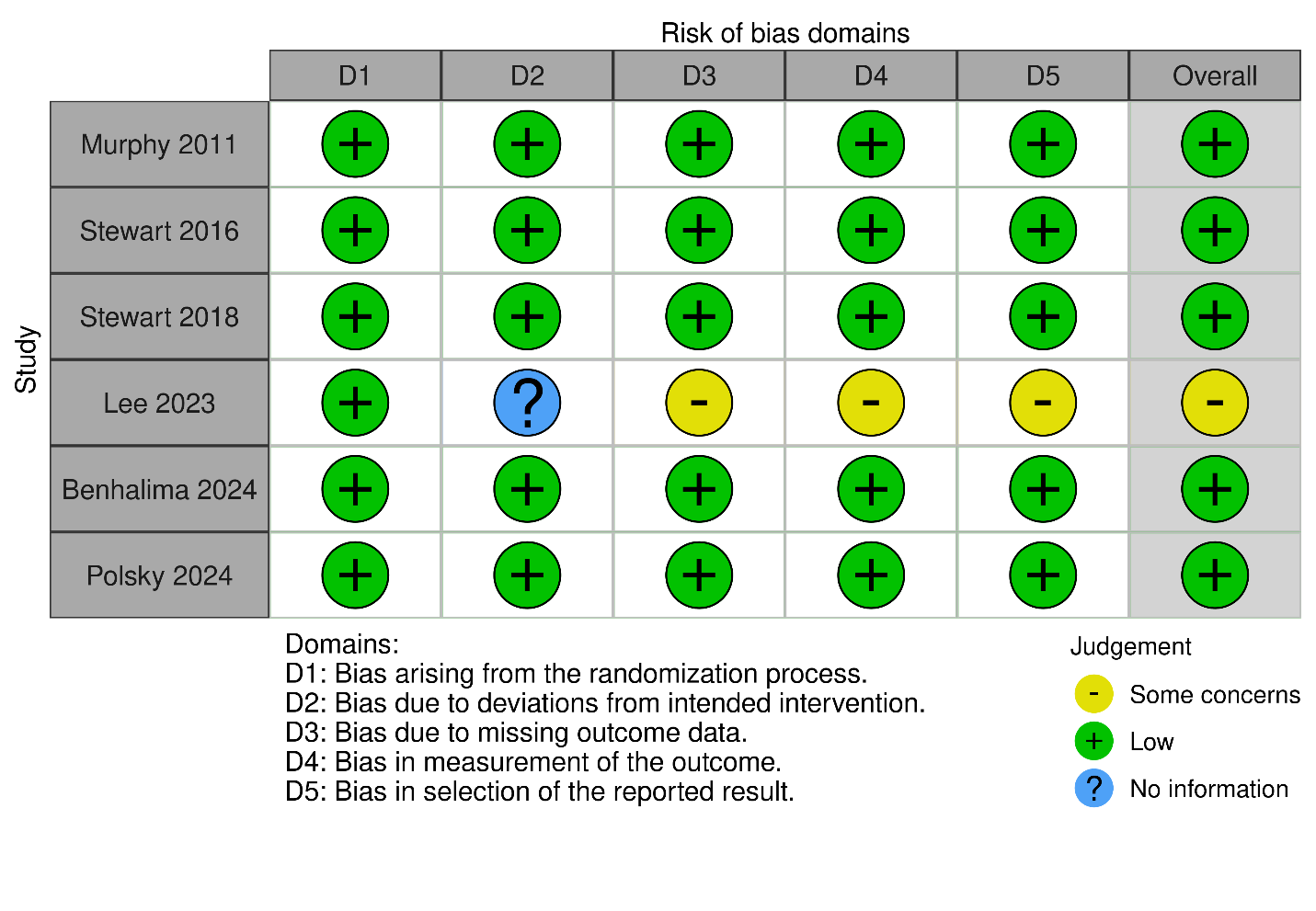
**

# **Figure S2 - Risk of bias (ROBINS-I) for observational studies**

Time in range (TIR) 63-140 mg/dl

**
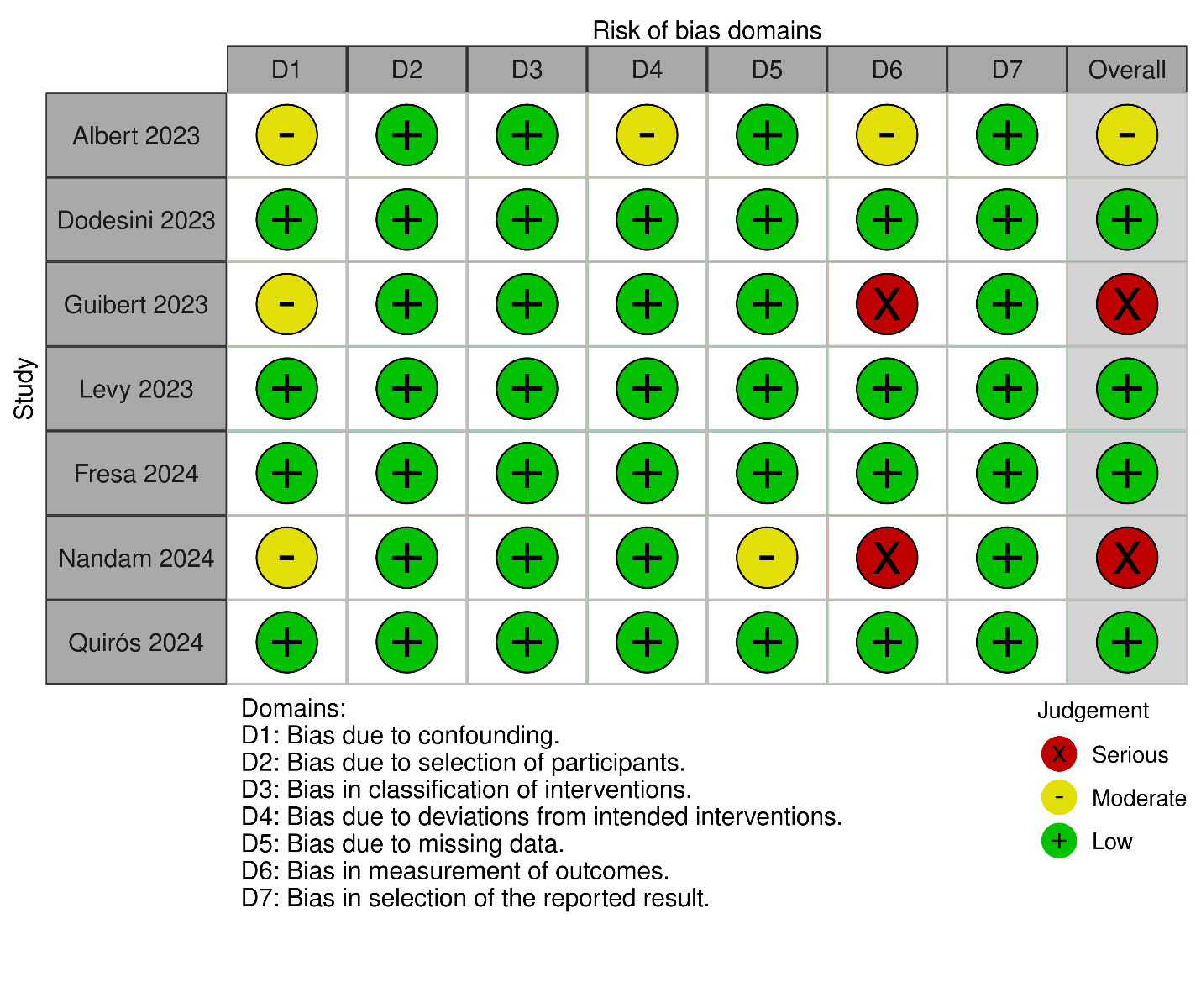
**

# **Table S2 – Certainty of evidence**

| Automated insulin delivery in pregnant women with type 1 diabetes mellitus: A systematic review and meta-analysis | | | | | | | | | | |
| --- | --- | --- | --- | --- | --- | --- | --- | --- | --- | --- |
| Quality assessment | | | | | | | **Summary of findings** | | | |
|  | | | | | | | **No of patient** | | **Effects** | **Quality** |
| No of studies | **Study design** | **Risk of Bias** | **Inconsistency** | **Indirectness** | **Imprecision** | **Other considerations** | **Advanced hybrid closed-loop system** | **Standard care** | **Absolute (95% CI)** |  |
| Percentage of time spent in the target glucose range (TIR) of 63-140 mg/dL | | | | | | | | | | |
| 6 | randomized | not serious | serious | not serious | not serious | none | 165 | 170 | MD 5.19 higher (0.06 to 10.32) | ⊕⊕⊕ Moderate |
| 7 | observational | not serious | not serious | not serious | not serious | none | 108 | 98 | MD 8.71 higher (4.51 to 12.90) | ⊕⊕ Low |
| Glycemic variability, % | | | | | | | | | | |
| 4 | randomized | not serious | not serious | not serious | not serious | none | 148 | 153 | MD -1.95 lower (-3.63 to -0.28) | ⊕⊕⊕⊕ High |
| 5 | observational | not serious | serious | not serious | serious | none | 98 | 88 | MD -0.89 lower (-2.16 to 0.38) | ⊕ Very low |
| Changes in glycated hemoglobin (HbA1c), % | | | | | | | | | | |
| 5 | randomized | not serious | serious | not serious | serious | none | 136 | 141 | MD 0.01 higher (-0.34 to 0.37) | ⊕⊕ Low |
| 6 | observational | not serious | serious | not serious | serious | none | 90 | 80 | MD -0.30 lower (-0.61 to 0.01) | ⊕ Very low |

# **Figure S3 - Forest plot of time spent in hypoglycaemia <63 mg/dl. Overall effect and subgroup analysis based on study design.**


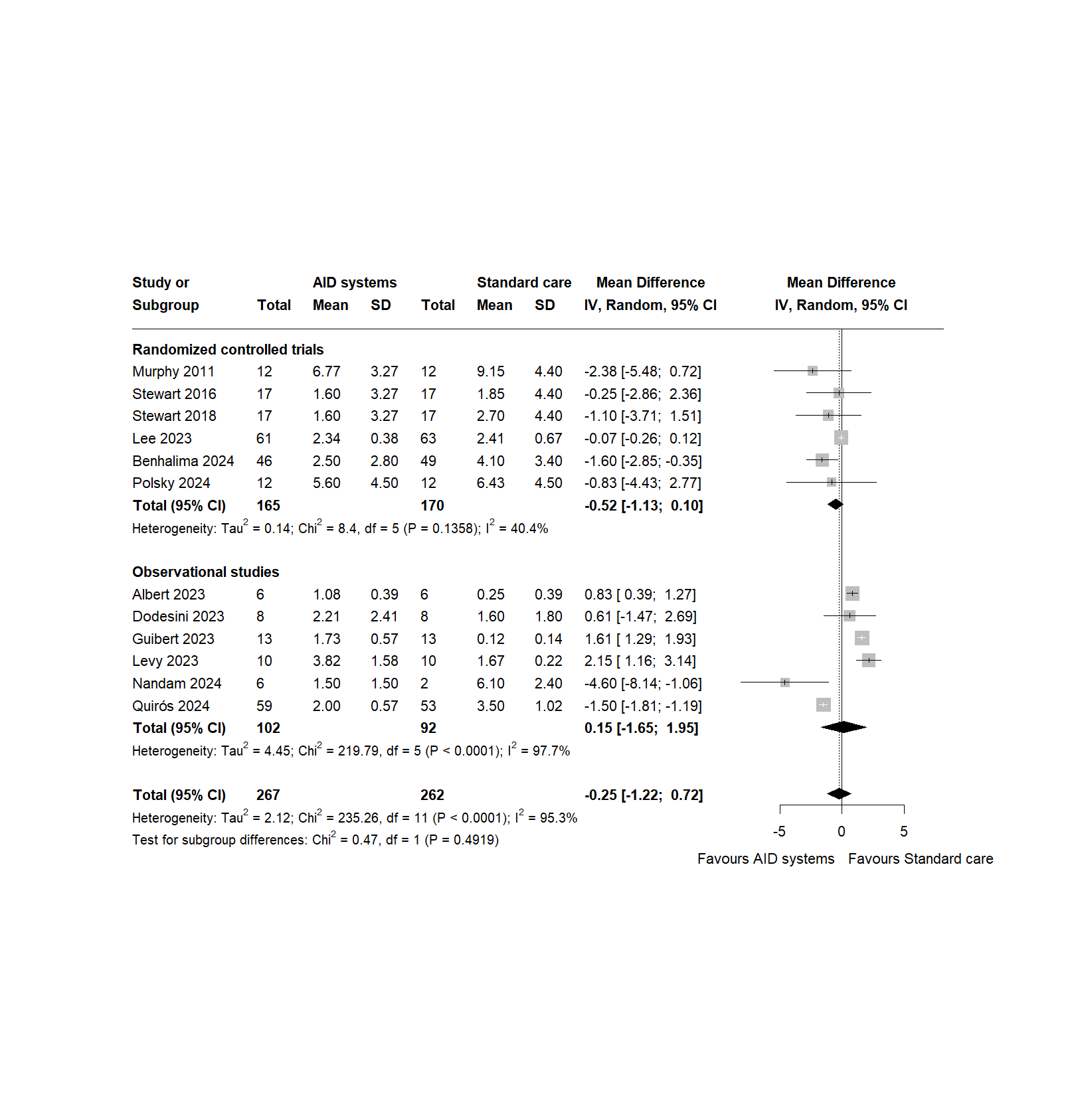


# **Figure S4 -** **Forest plot of** **time spent in hypoglycaemia <54 mg/dl. Overall effect and subgroup analysis based on study design.**


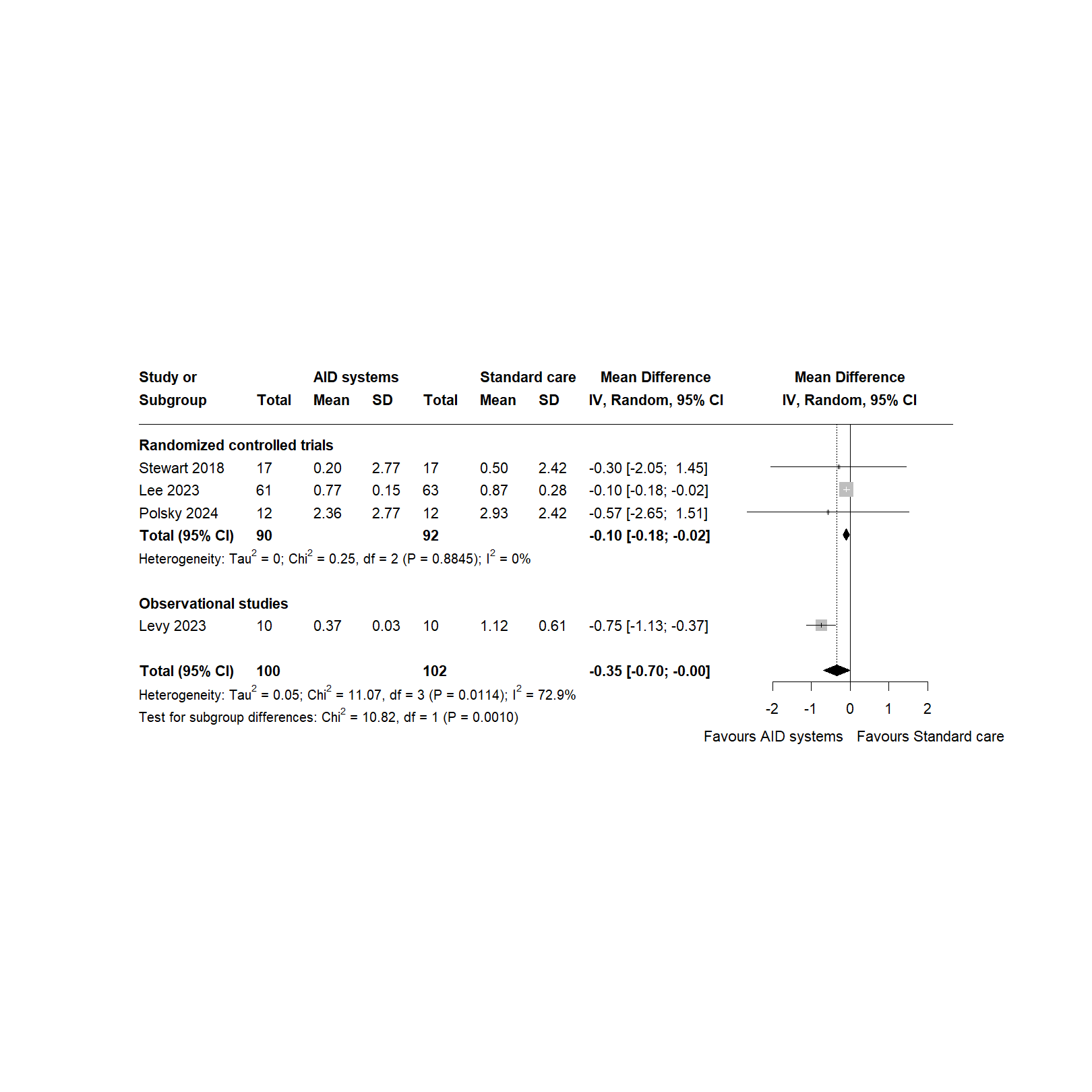


# **Figure S5 - Forest plot of time spent in hyperglycaemia >180 mg/dl. Overall effect and subgroup effects based on study design.**


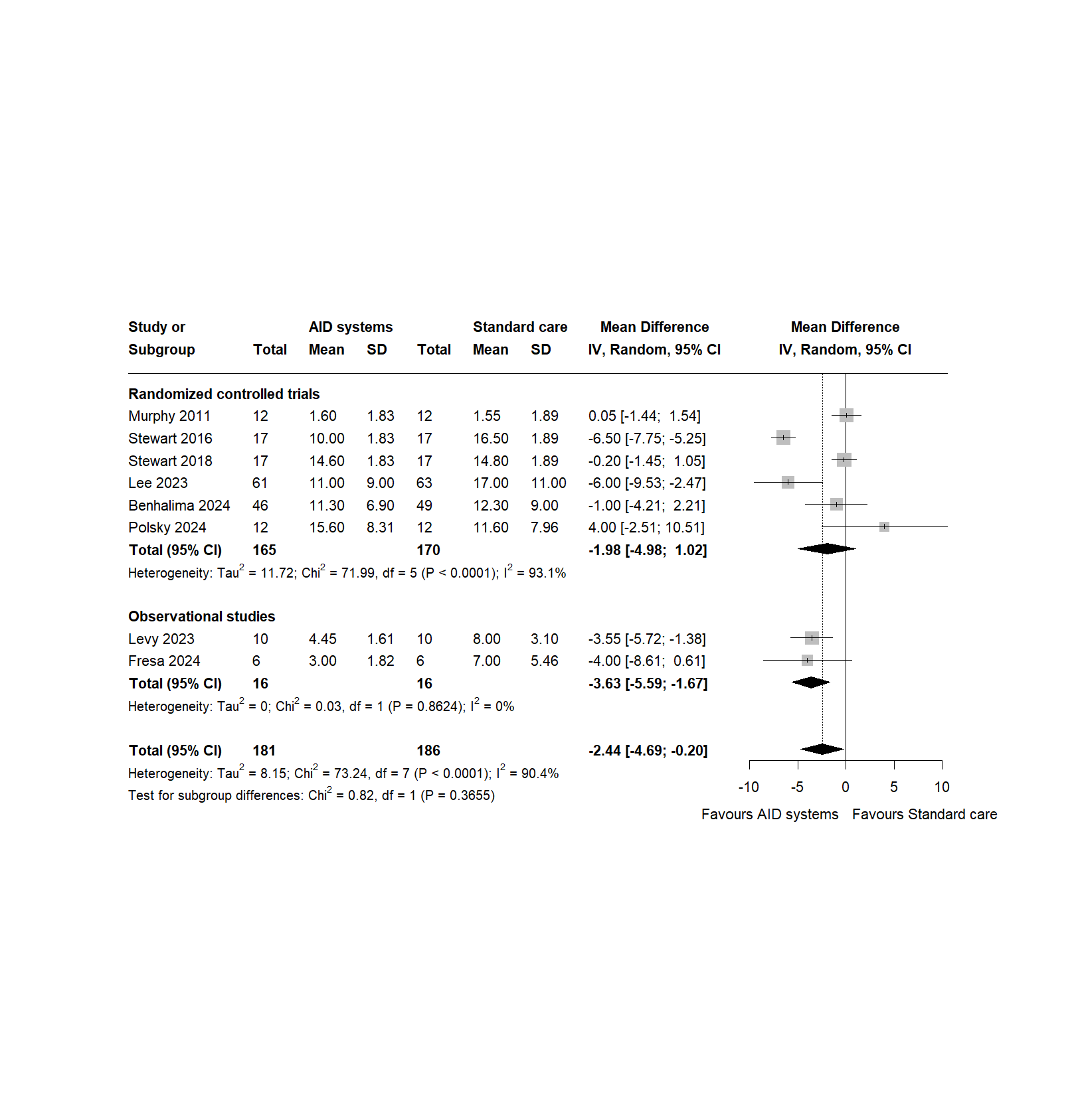


# **Figure S6 - Forest plot of time in range (TIR, 24-h percentage) 63-140 mg/dl after excluding studies with moderate or high risk of bias.**


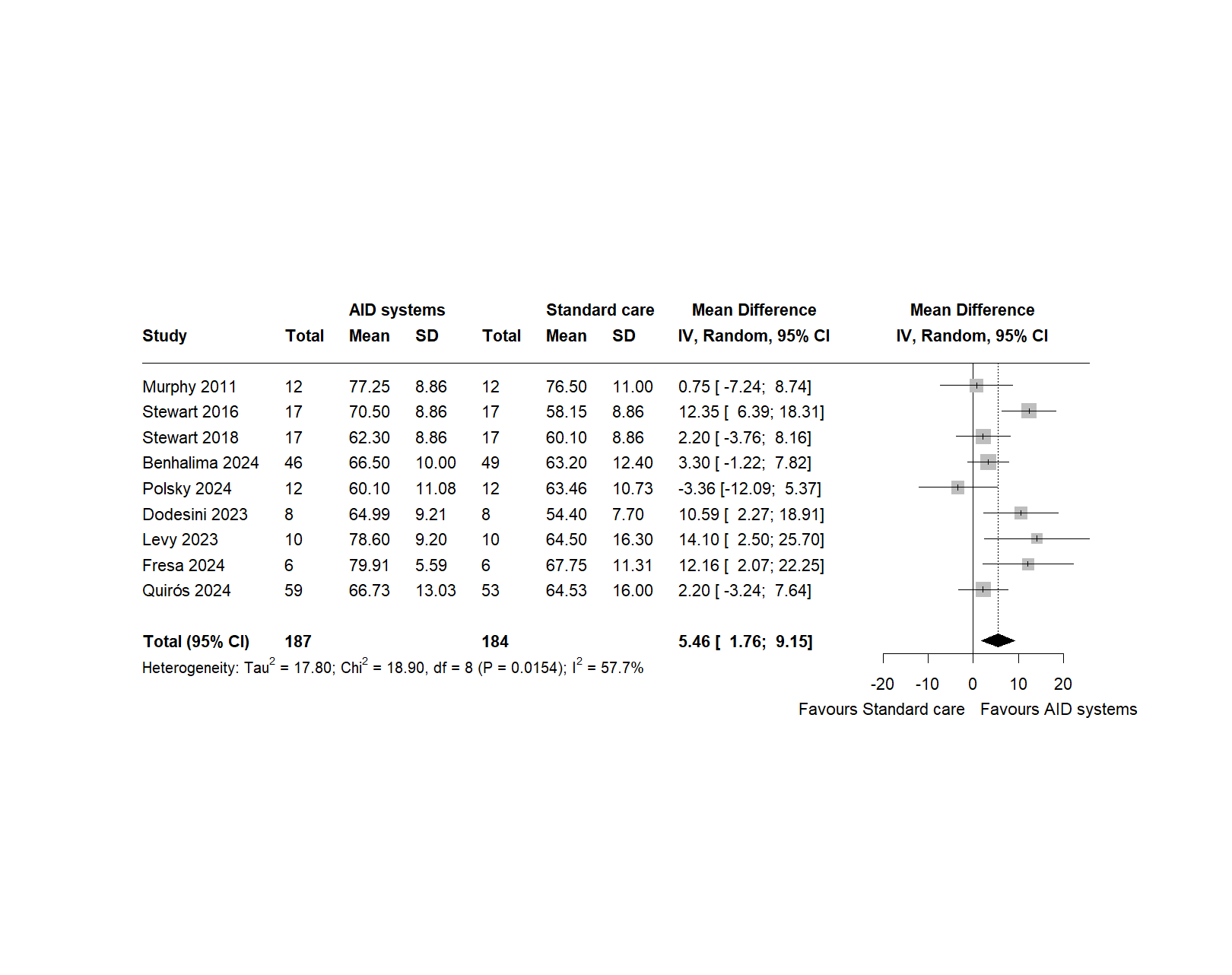


**Figure S7 - Forest plot of time spent in hypoglycaemia <54 mg/dl after excluding studies with moderate or high risk of bias.**

**
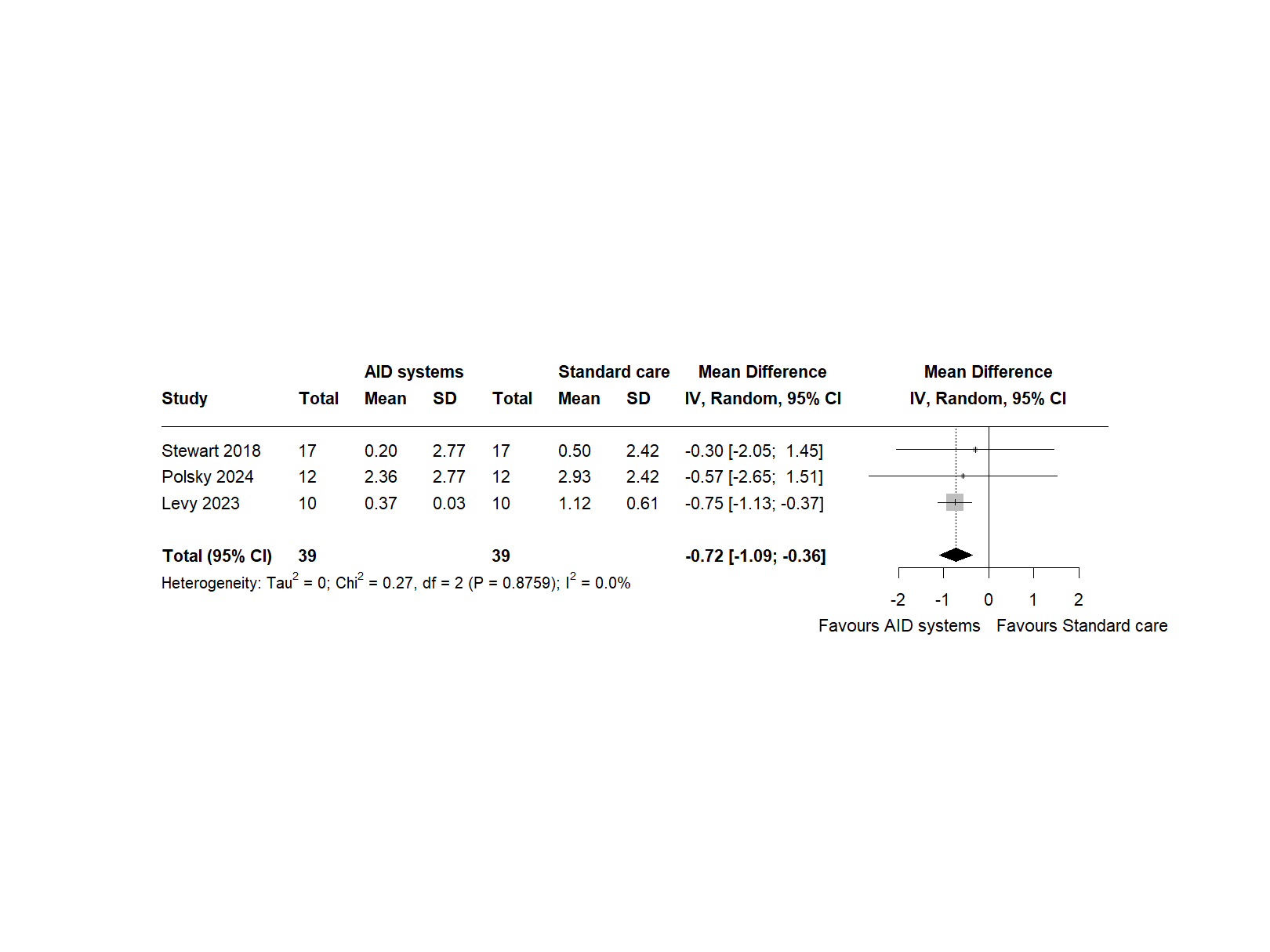
**

# **Figure S8 - Forest plot of changes in HbA1c (%). Overall effect and subgroup analysis based on study design.**


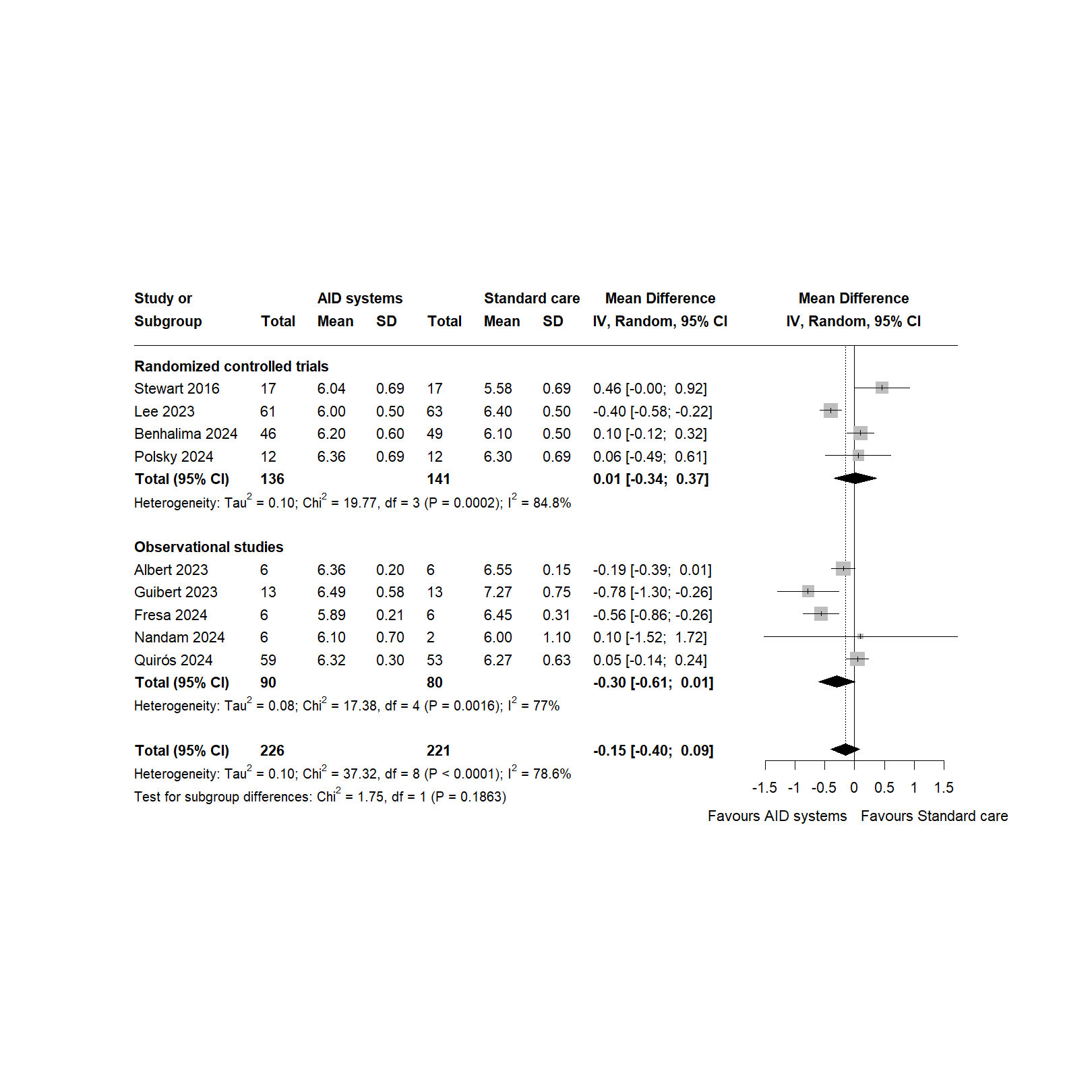


**Figure S9** **- Forest plot of glycaemic variability (CV, %) after excluding studies with moderate or high risk of bias.**


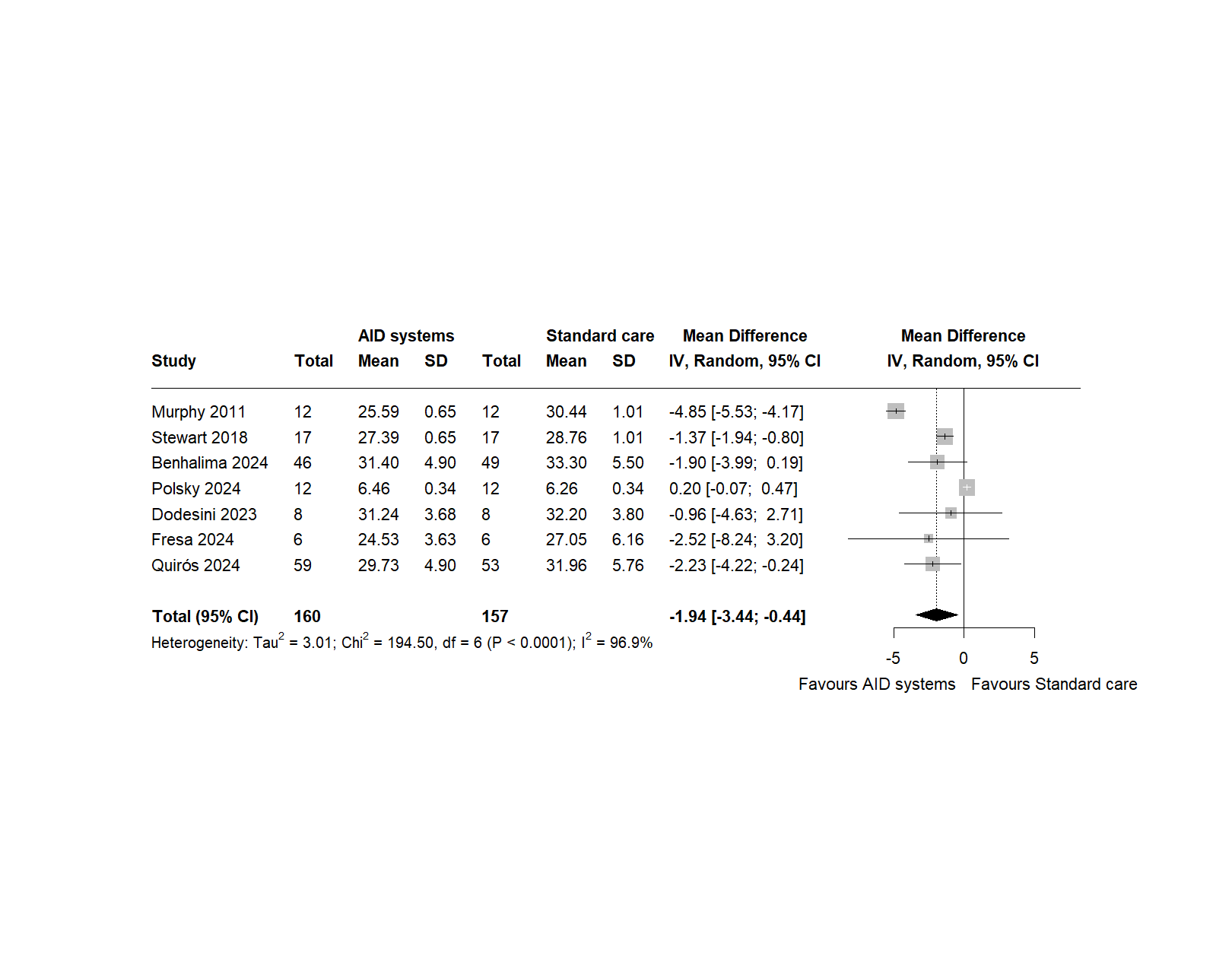


# **Figure S10 - Forest plot of changes in HbA1c (%) after excluding studies with moderate or high risk of bias.**


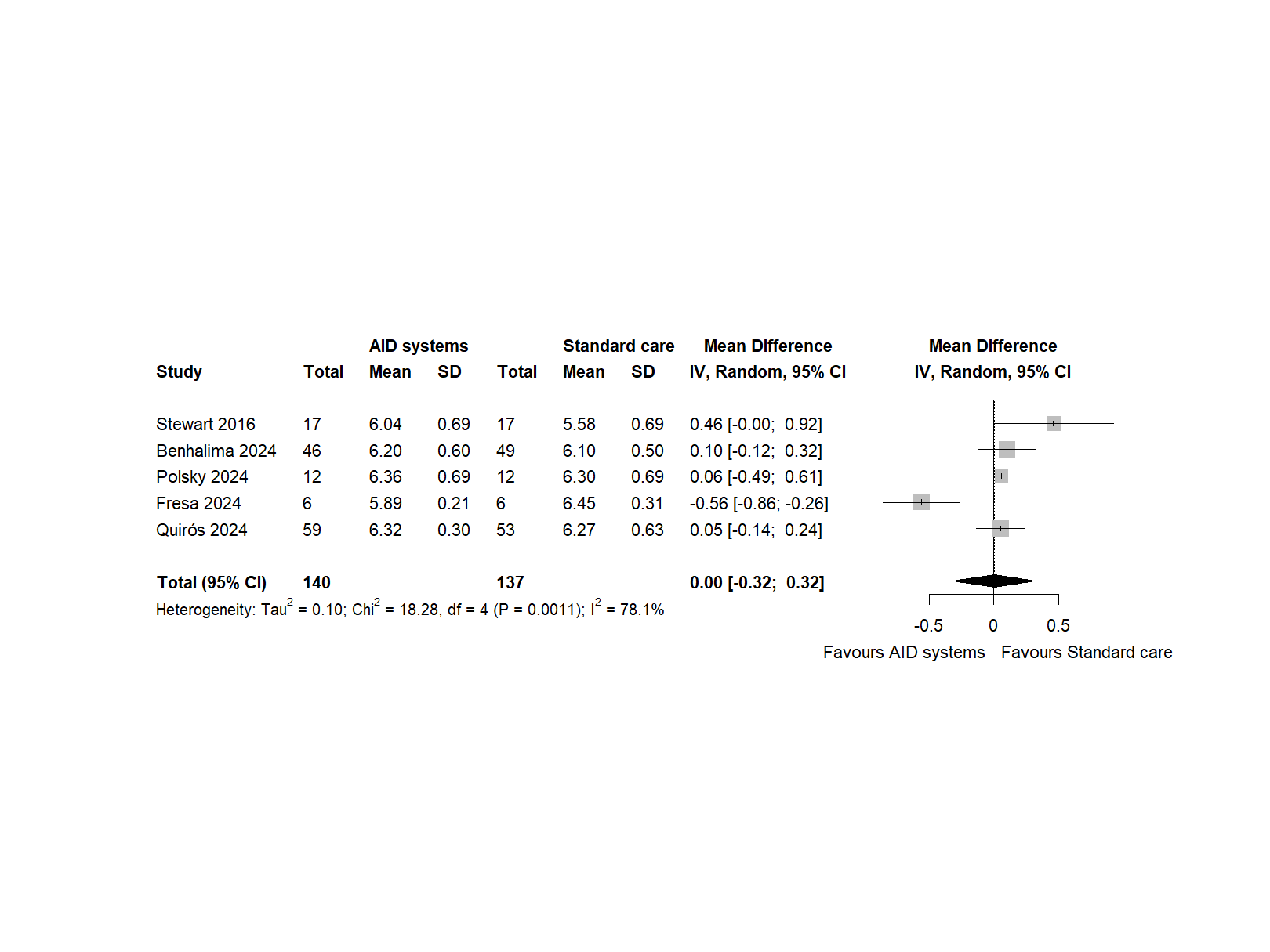


# **Figure S11 - Forest plot of total daily insulin dose (units/day/kg). Overall effect and subgroup analysis based on study design.**


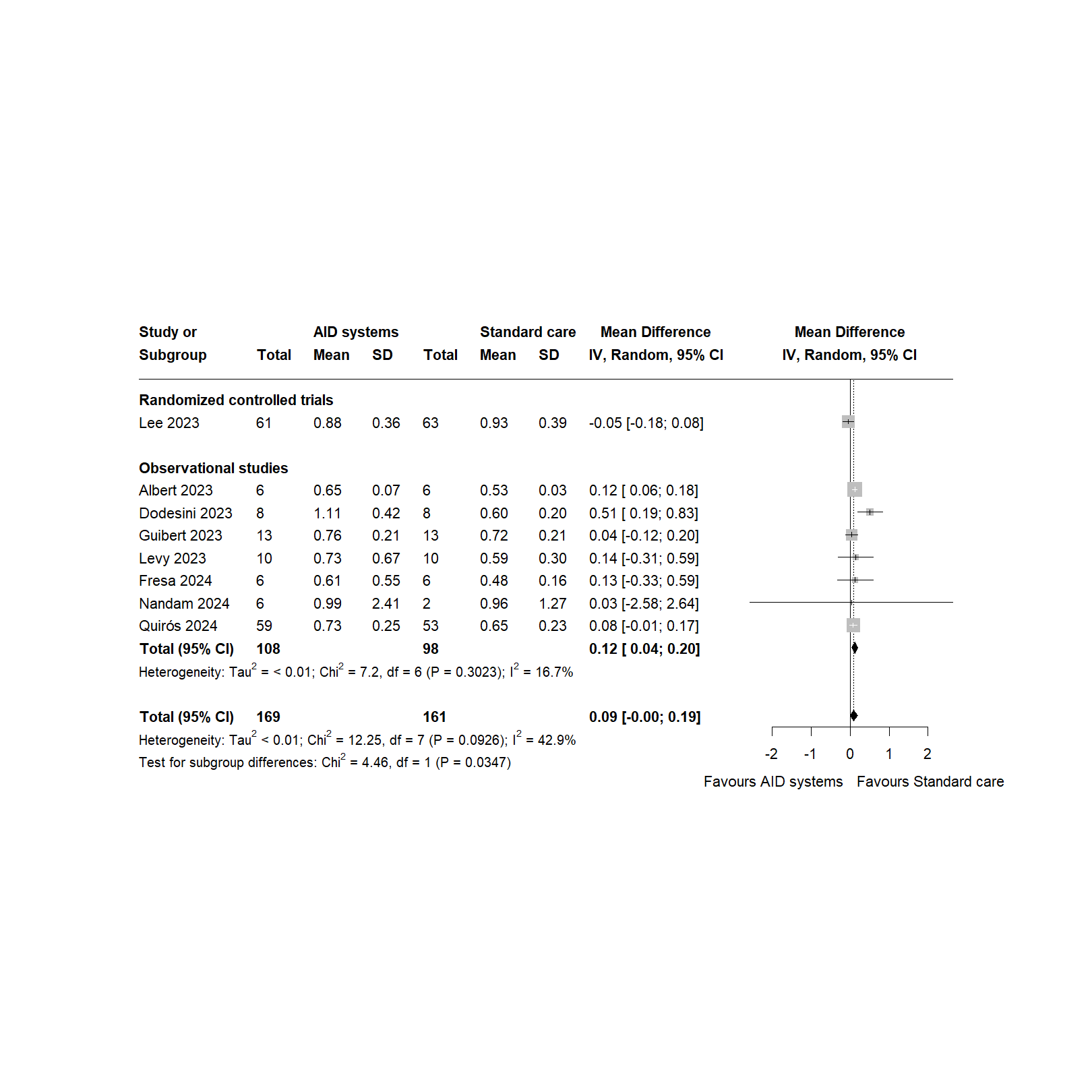


# **Figure S12 - Forest plot of total daily insulin dose (units/day/kg) after excluding studies with moderate or high risk of bias.**


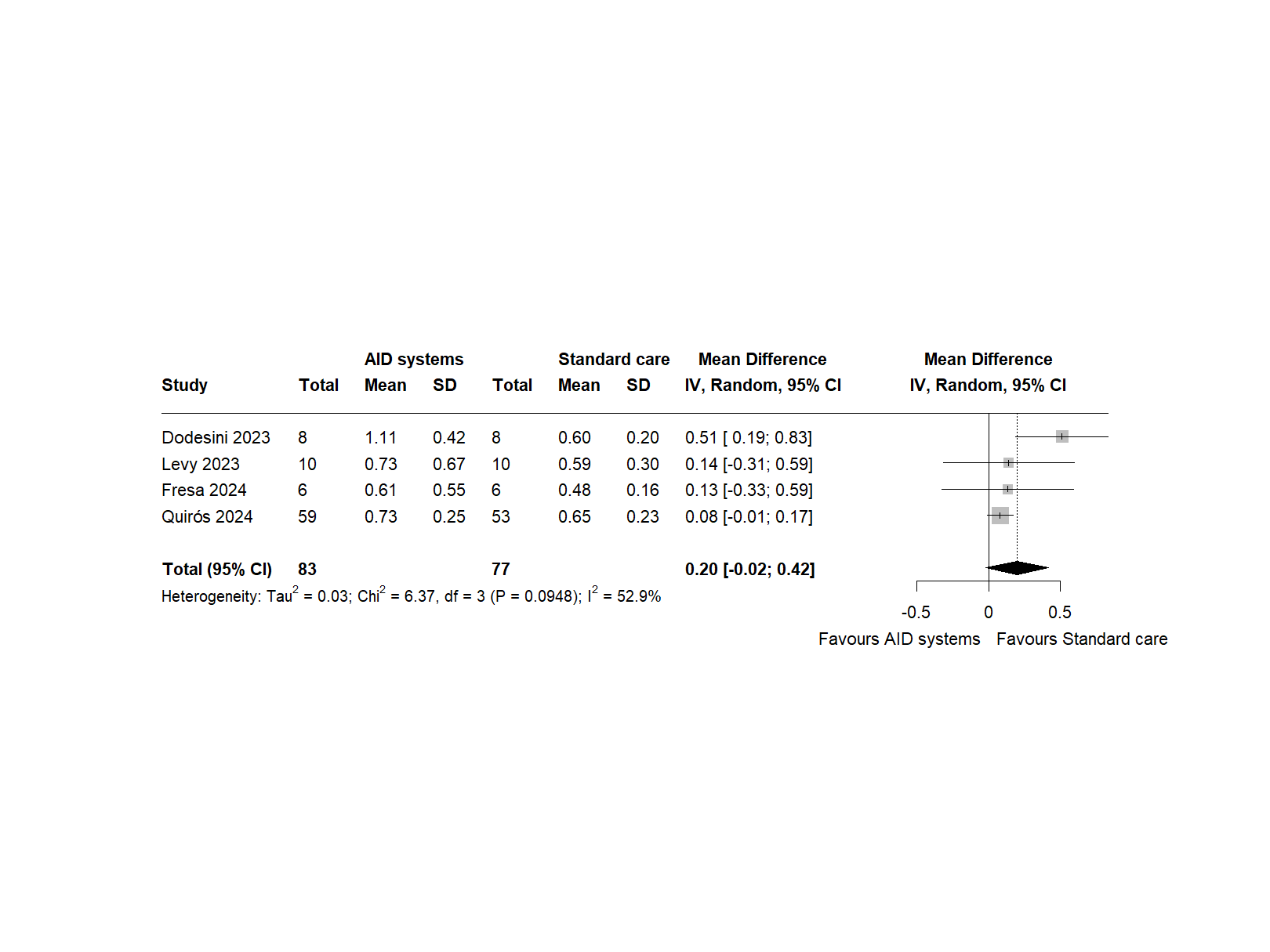


# **Figure S13 - Forest plot of severe hypoglycaemia. Overall effect and subgroup analysis based on study design.**


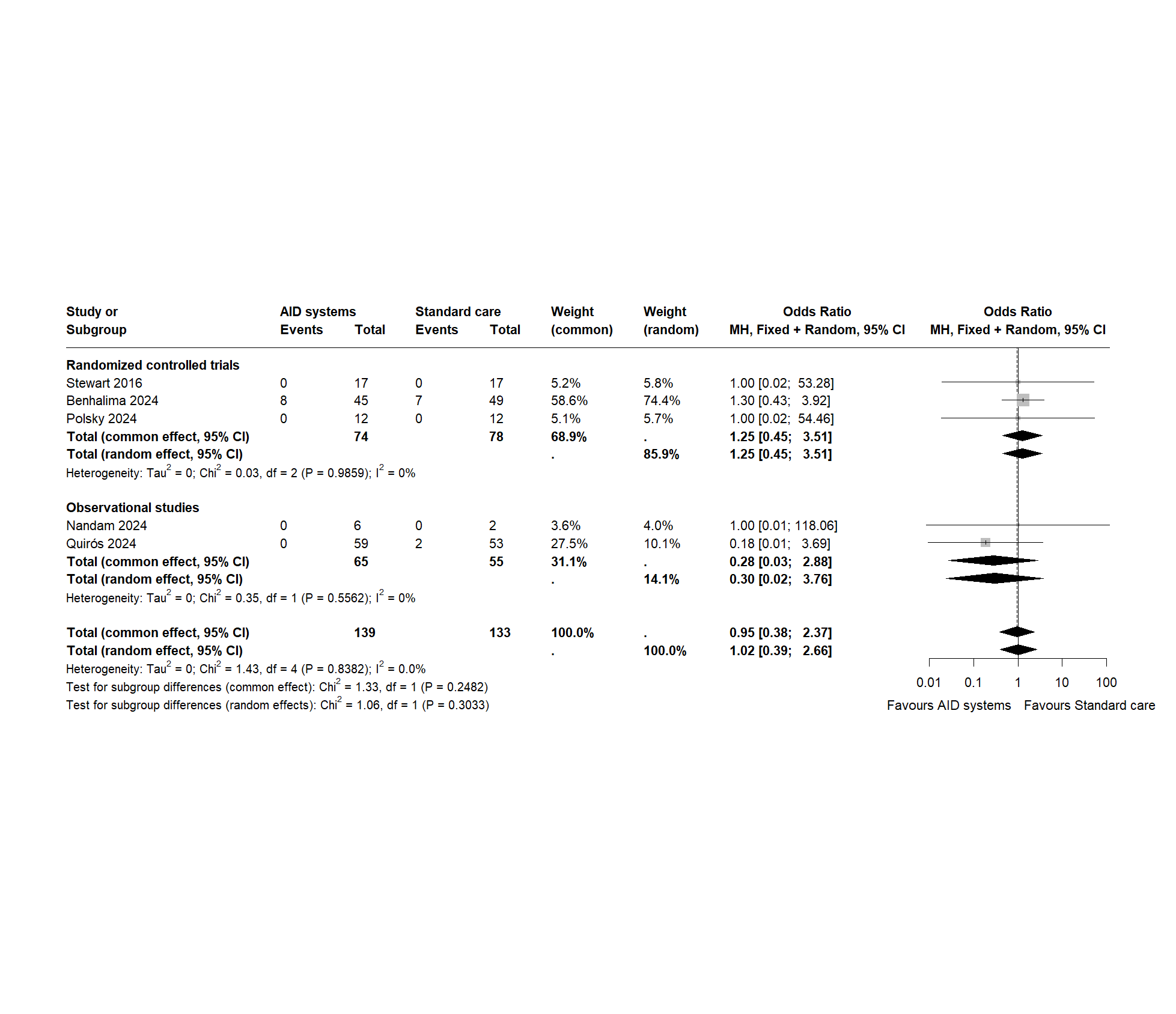


# **Figure S14 - Forest plot of gestational weight gain (kg). Overall effect and subgroup analysis based on study design.**


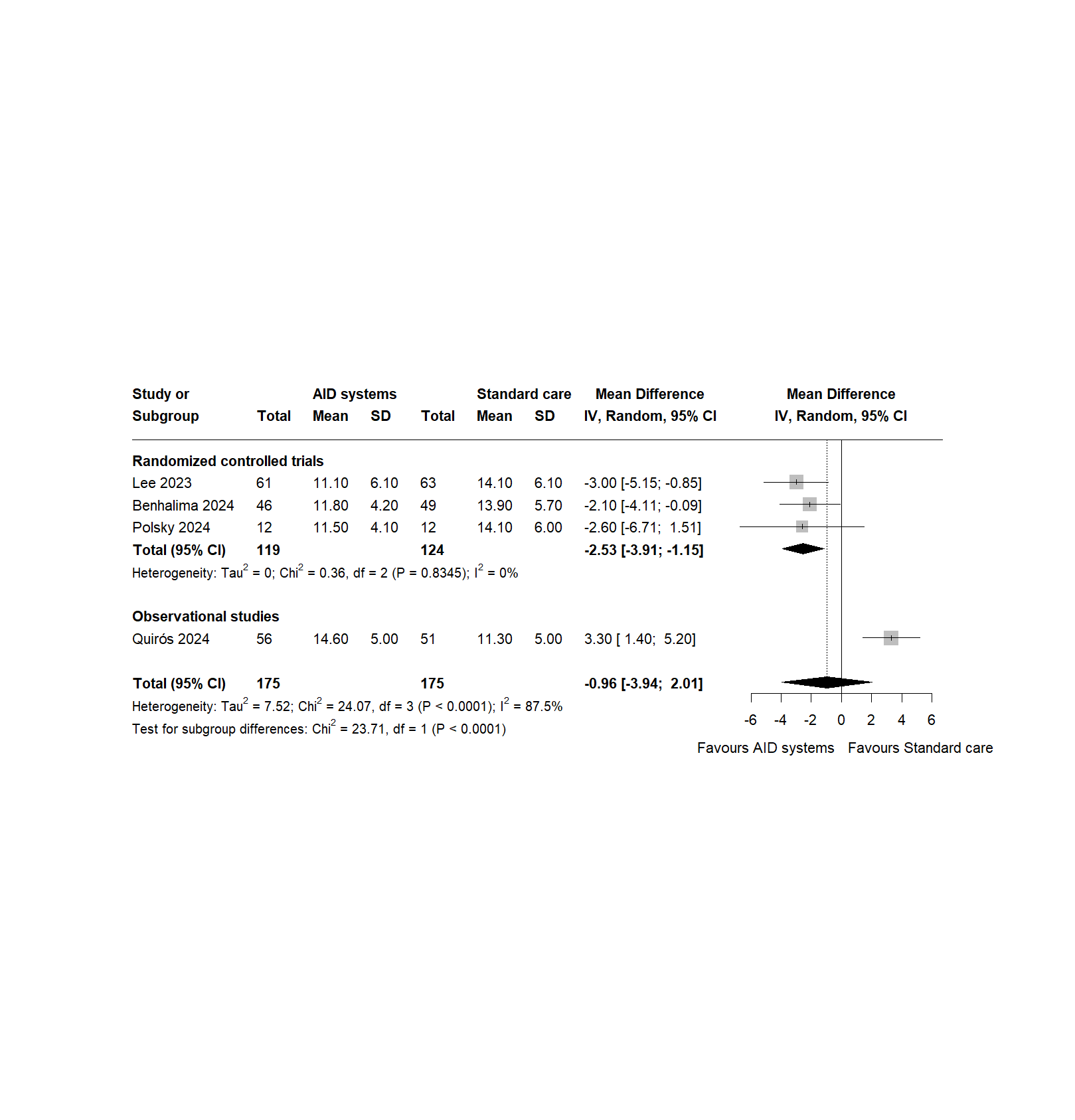


# **Figure S15 - Forest plot of incidence of patients suffered from preeclampsia. Overall effect and subgroup analysis based on study design.**


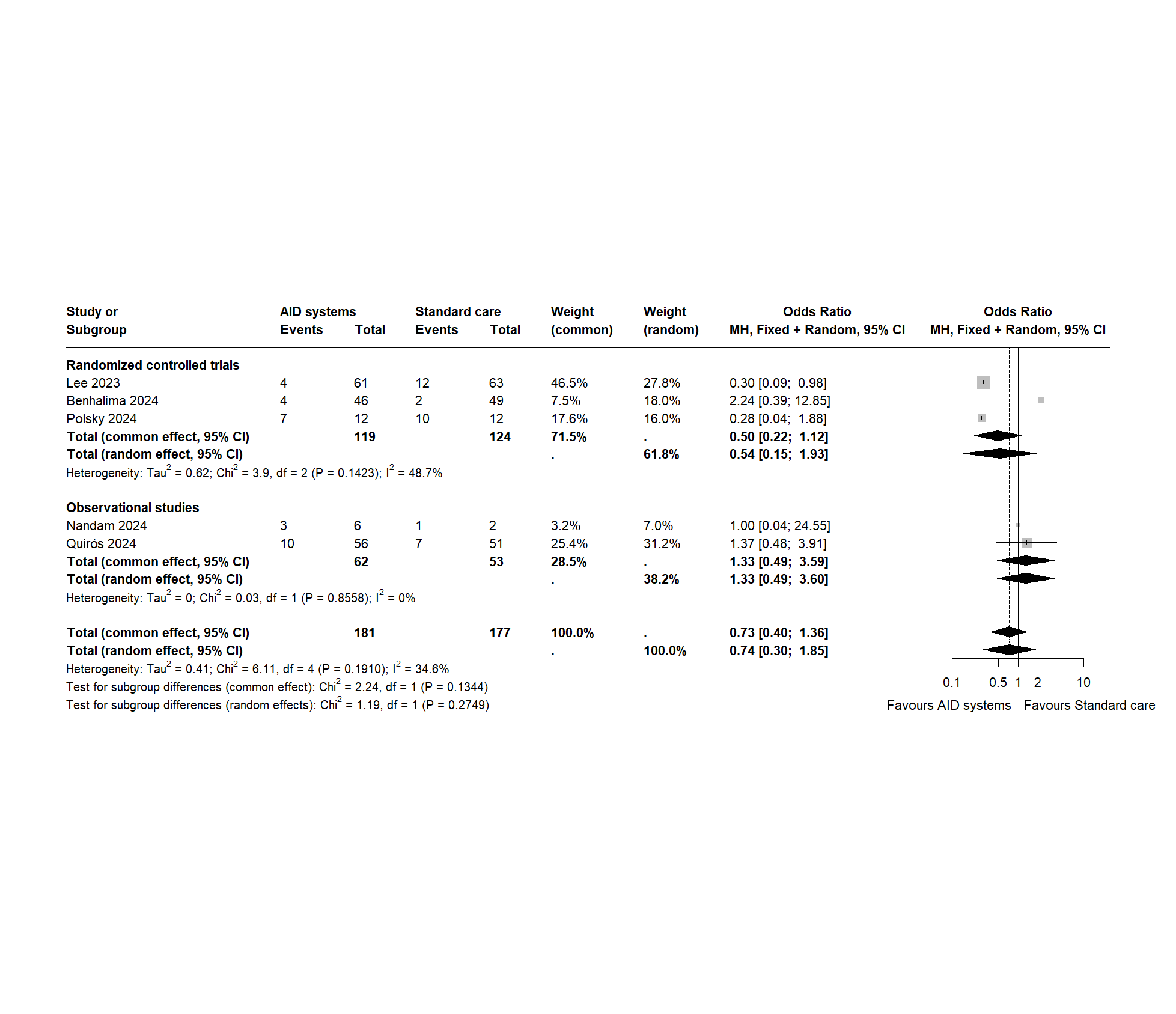


# **Figure S16 - Forest plot of proportion of cesarean delivery. Overall effect and subgroup analysis based on study design.**


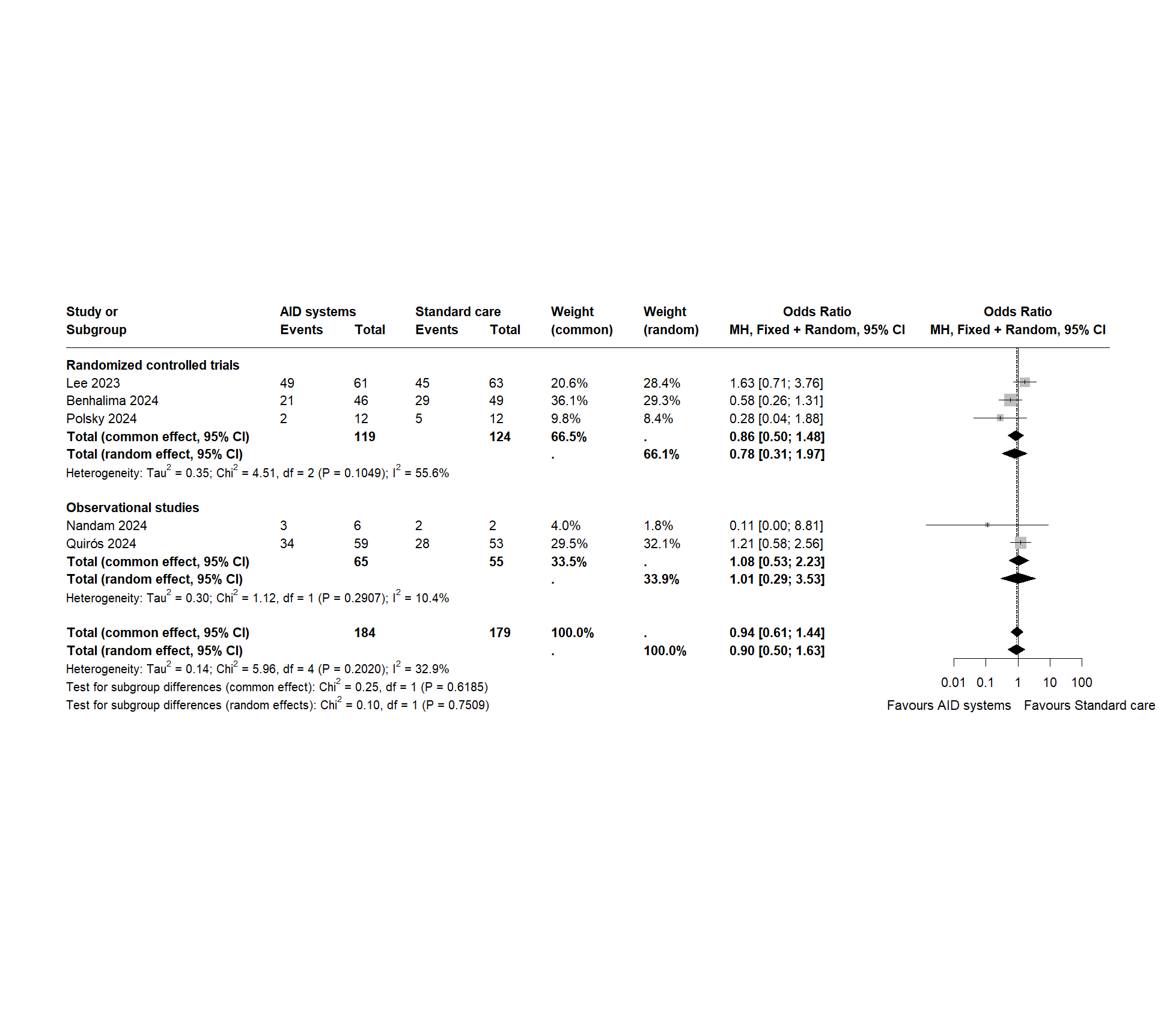


# **Figure S17 - Forest plot of birth weight (g). Overall effect and subgroup analysis based on study design.**


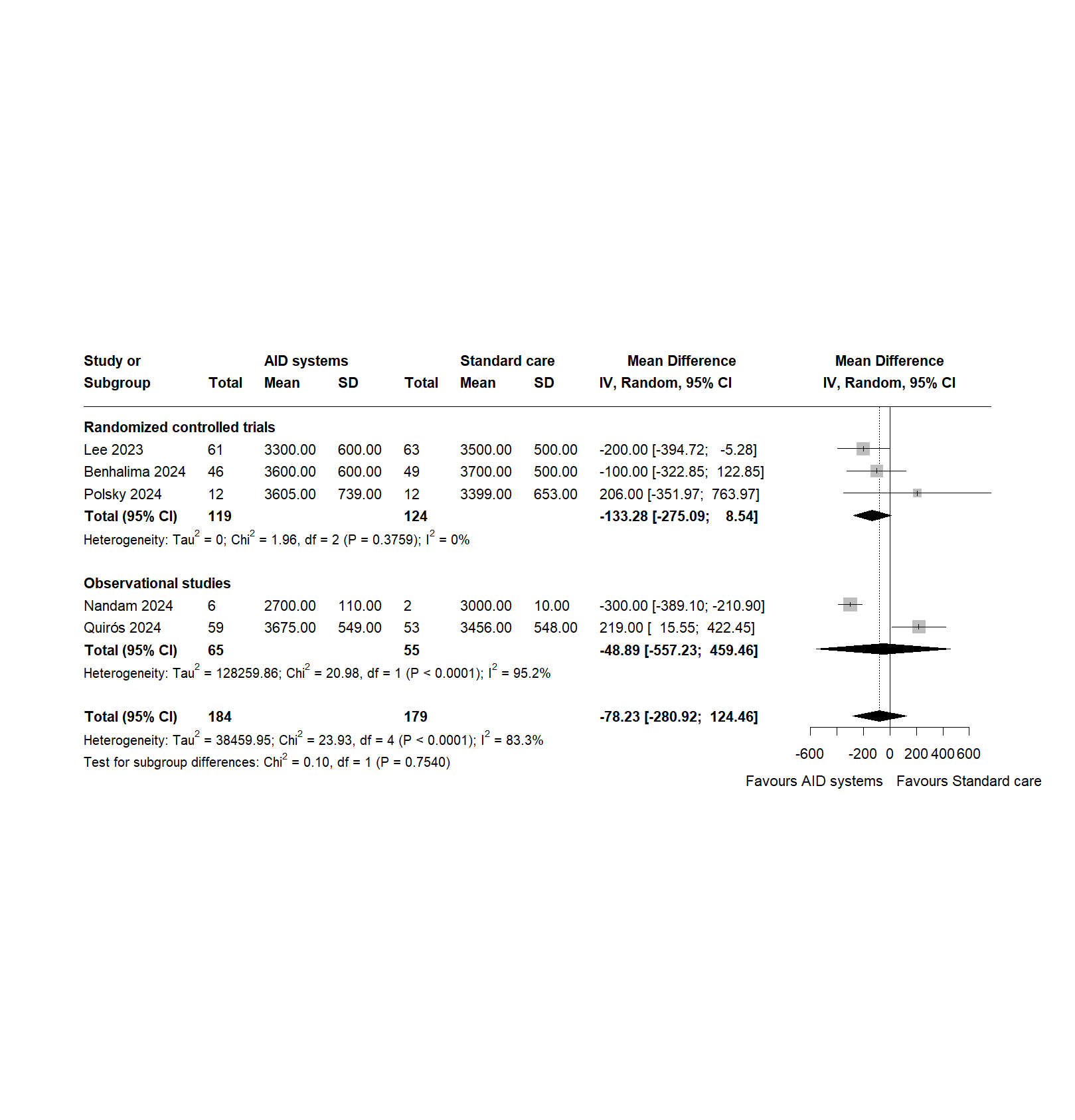


# **Figure S18 - Forest plot of incidence of neonates born with macrosomia >4gr. Overall effect and subgroup analysis based on study design.**


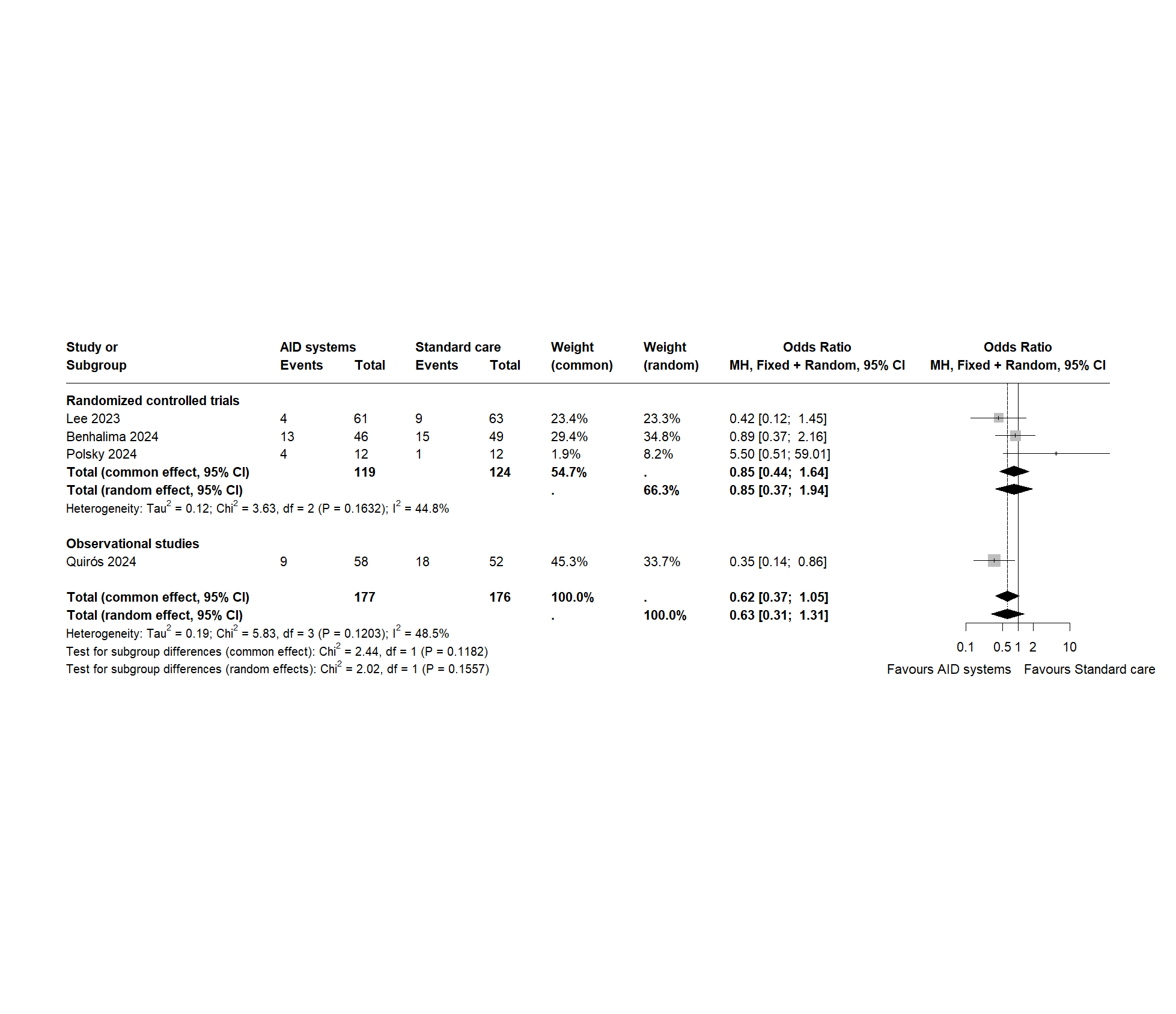


# **Figure S19 - Forest plot of incidence of LGA neonates. Overall effect and subgroup analysis based on study design.**


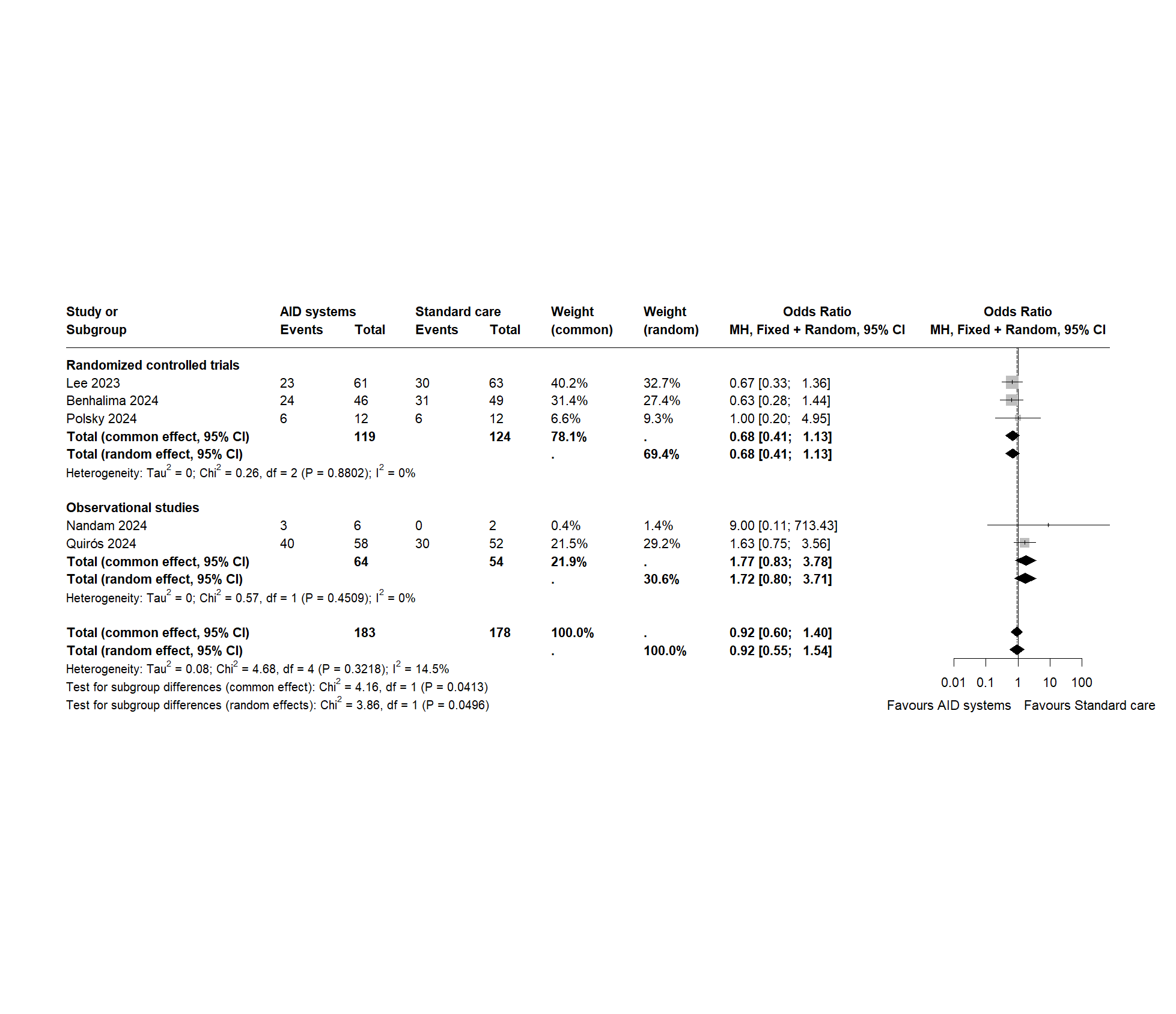


# **Figure S20 - Forest plot of incidence of SGA neonates. Overall effect and subgroup analysis based on study design.**


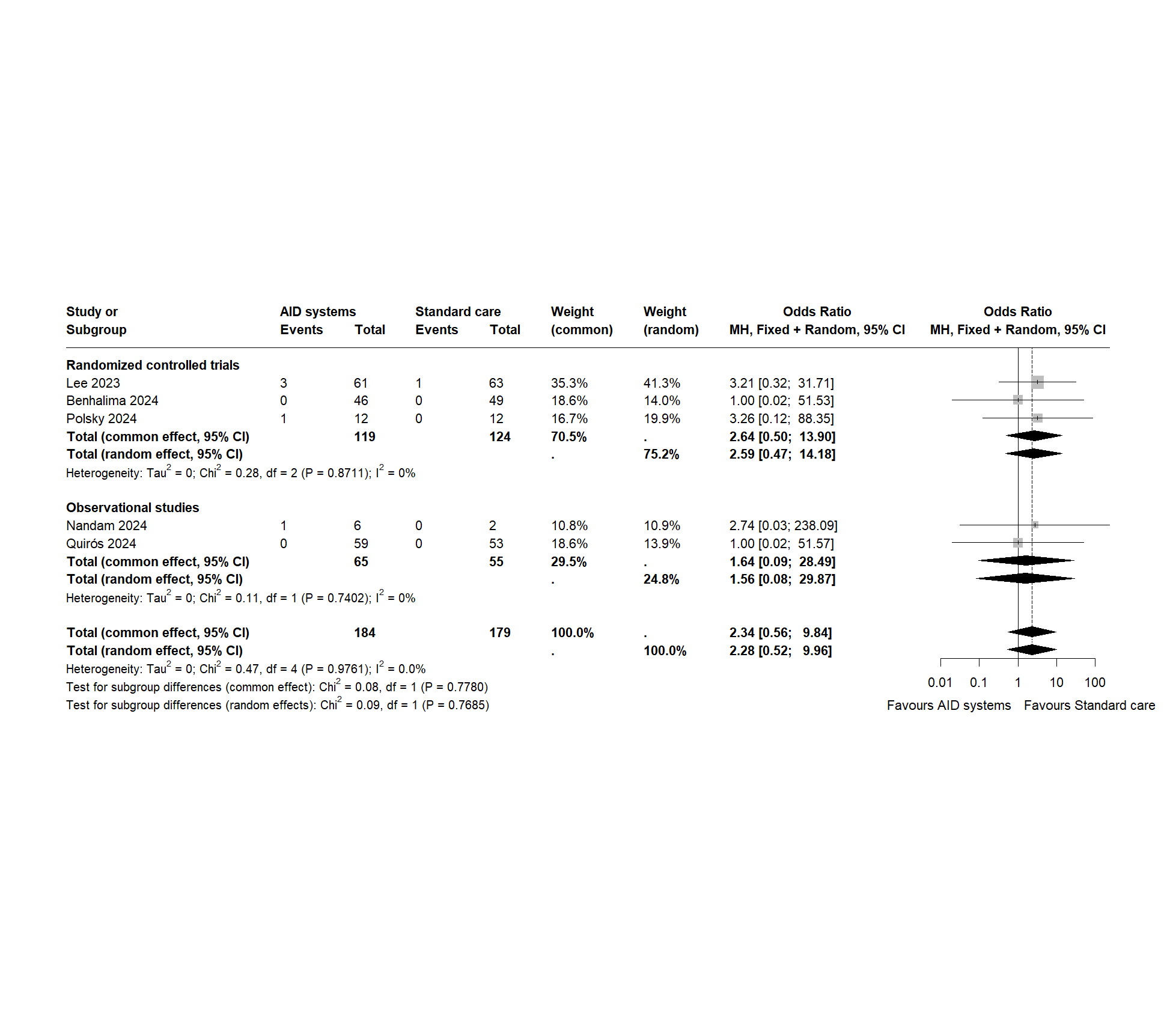


# **Figure S21 - Forest plot of proportion of NICY admissions (>24h). Overall effect and subgroup analysis based on study design.**


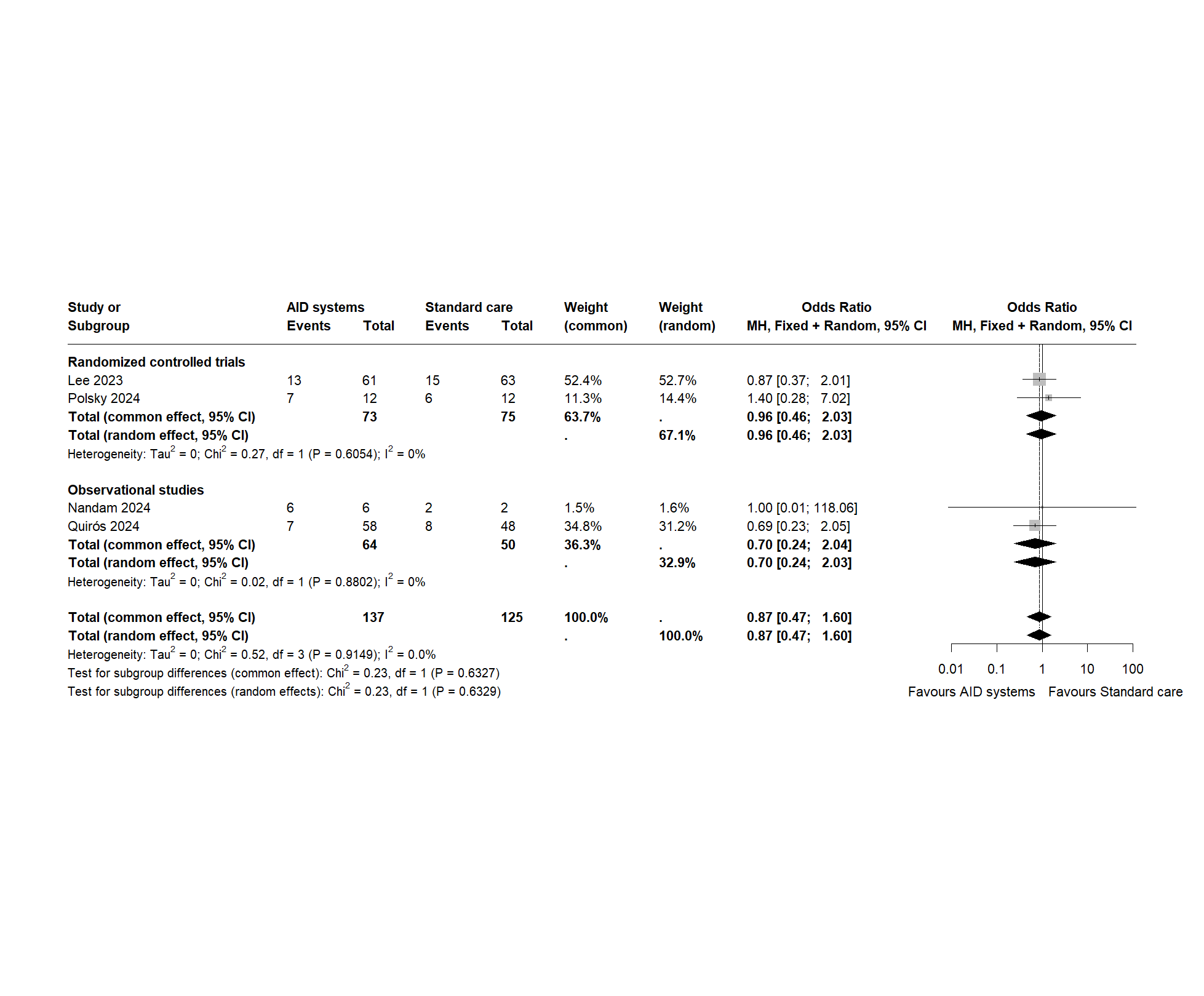


# **Figure S22 - Forest plot of duration of NICY admissions (days). Overall effect.**


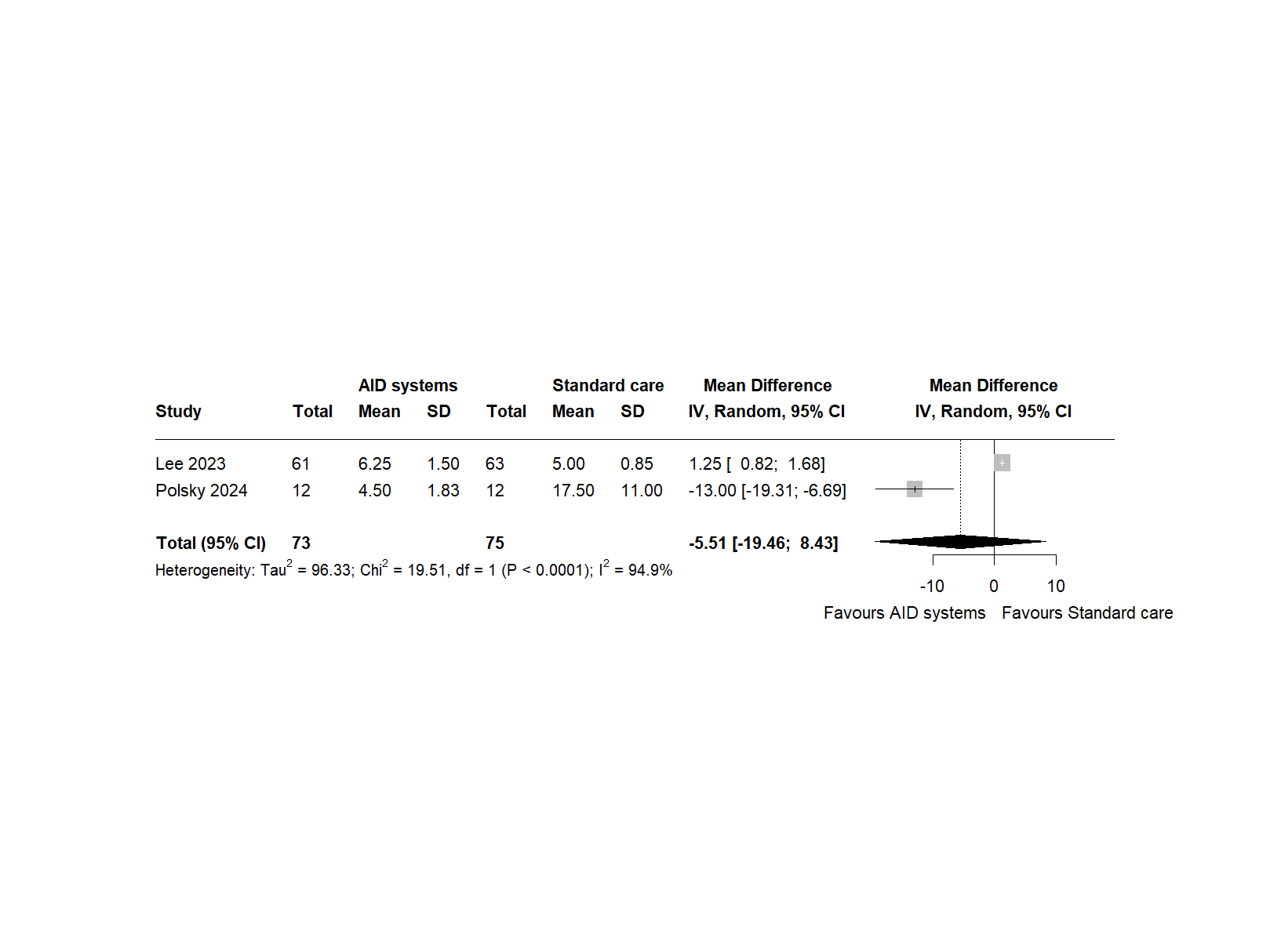


# **Figure S23 - Forest plot of incidence for preterm delivery. Overall effect and subgroup analysis based on study design.**


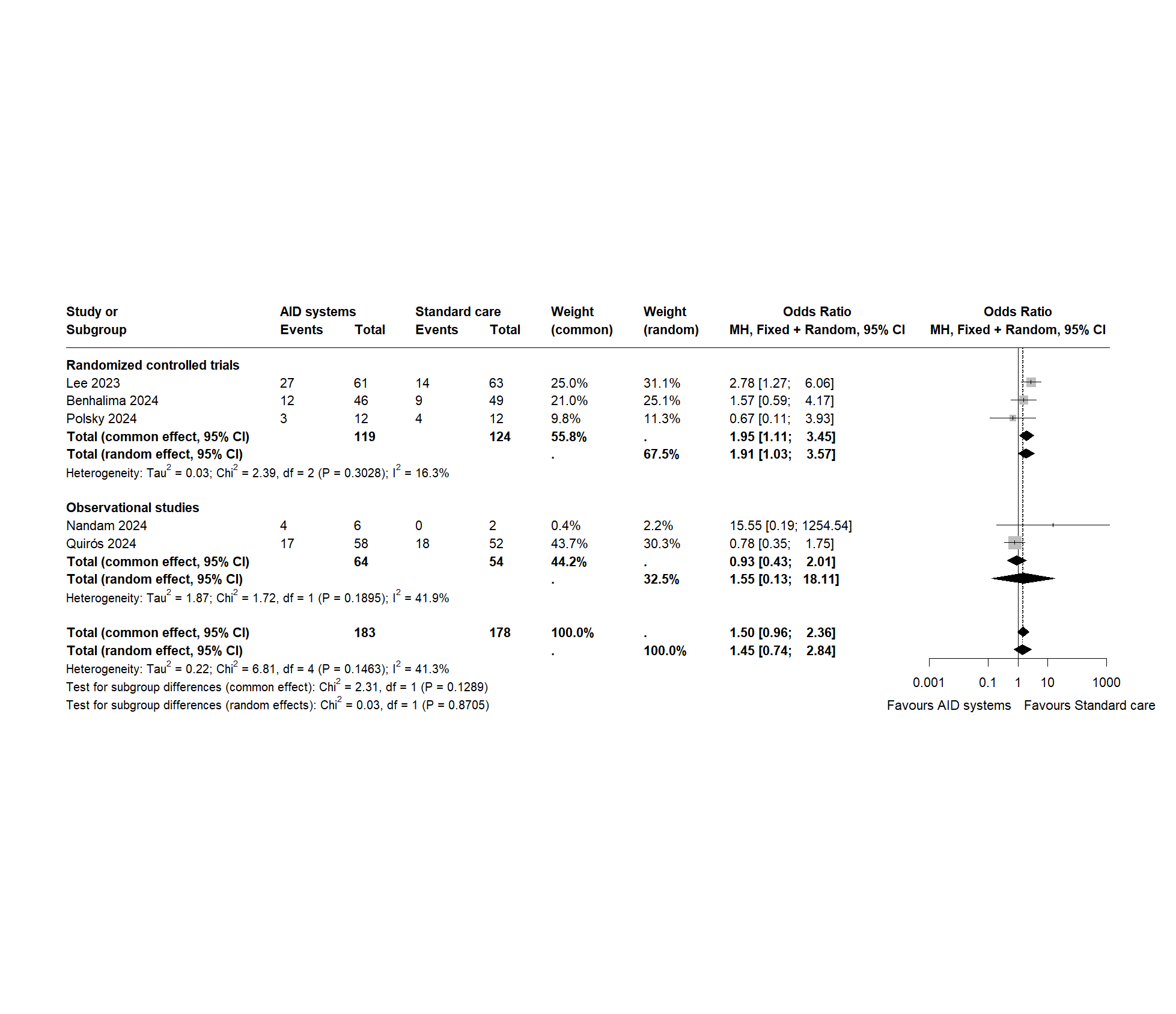


# **Figure S24 - Forest plot of hypoglycaemia requiring glycose. Overall effect.**


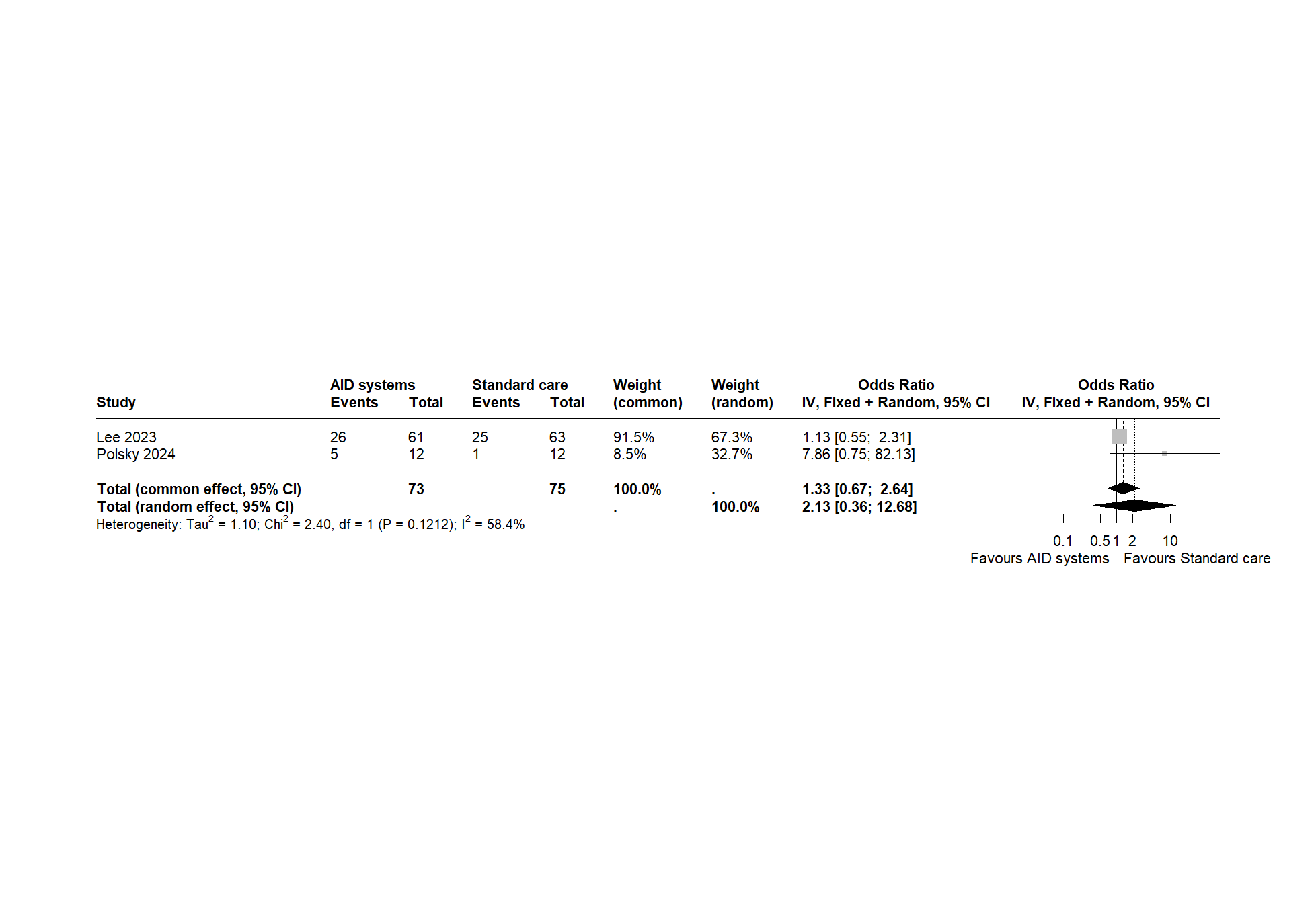


# **Figure S25 - Forest plot of still or neonatal deaths. Overall effect and subgroup analysis based on study design.**


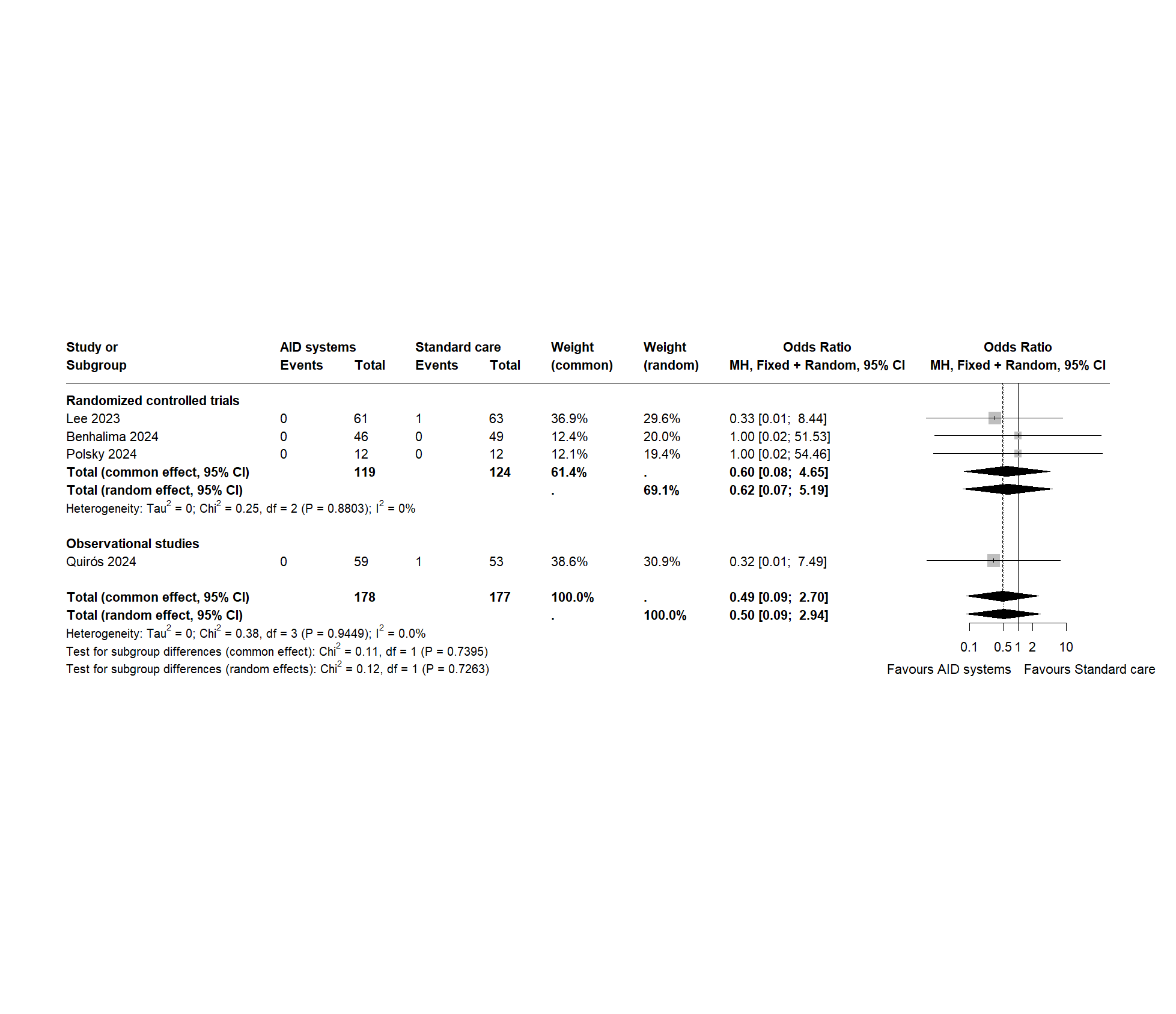


# **Figure S26 - Forest plot of time spent in range 63-140 mg/dl during the 1^st^ trimester. Overall effect.**


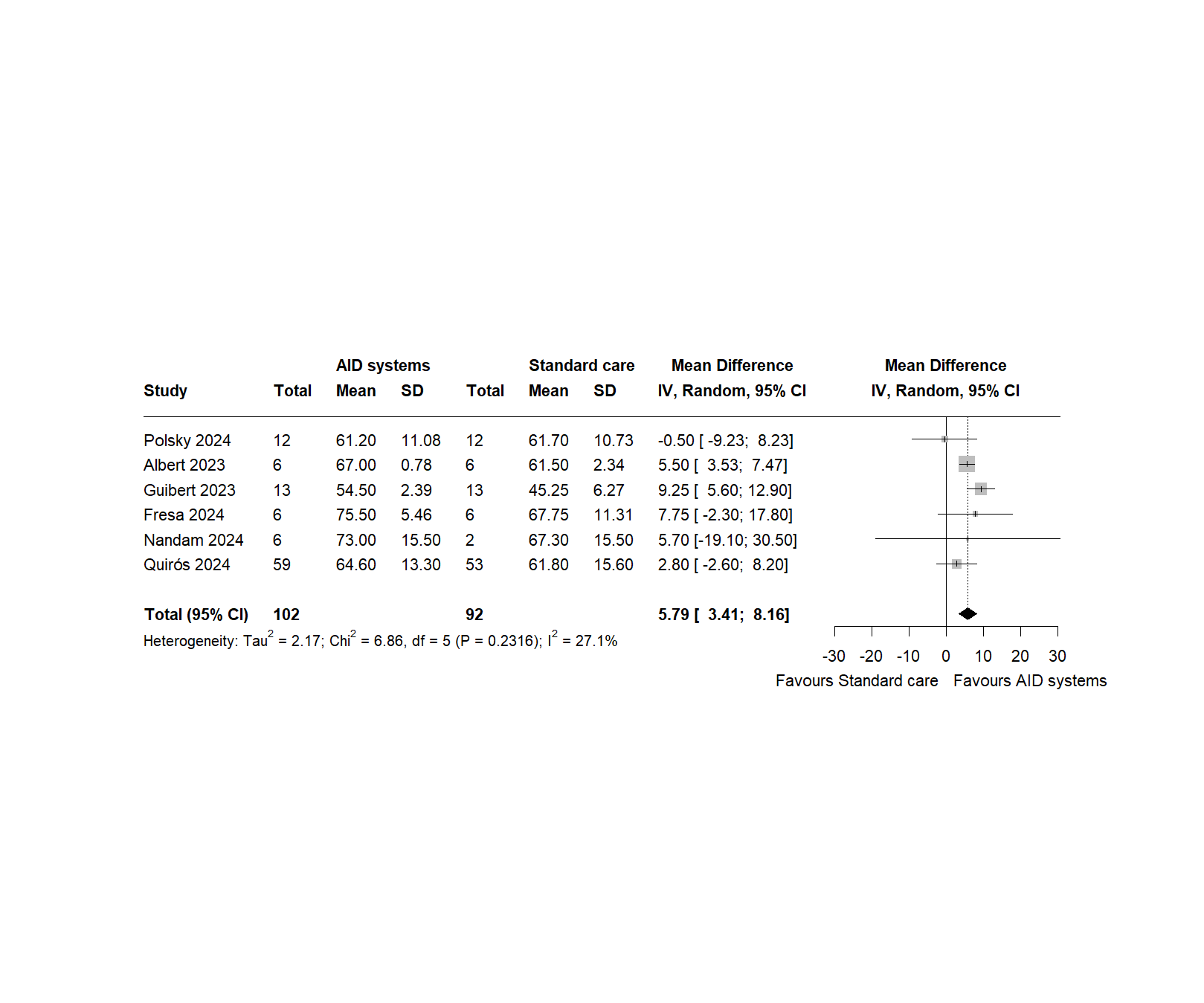


# **Figure S27 - Forest plot of time spent in hyperglycaemia >140 mg/dl during the 1^st^ trimester. Overall effect.**


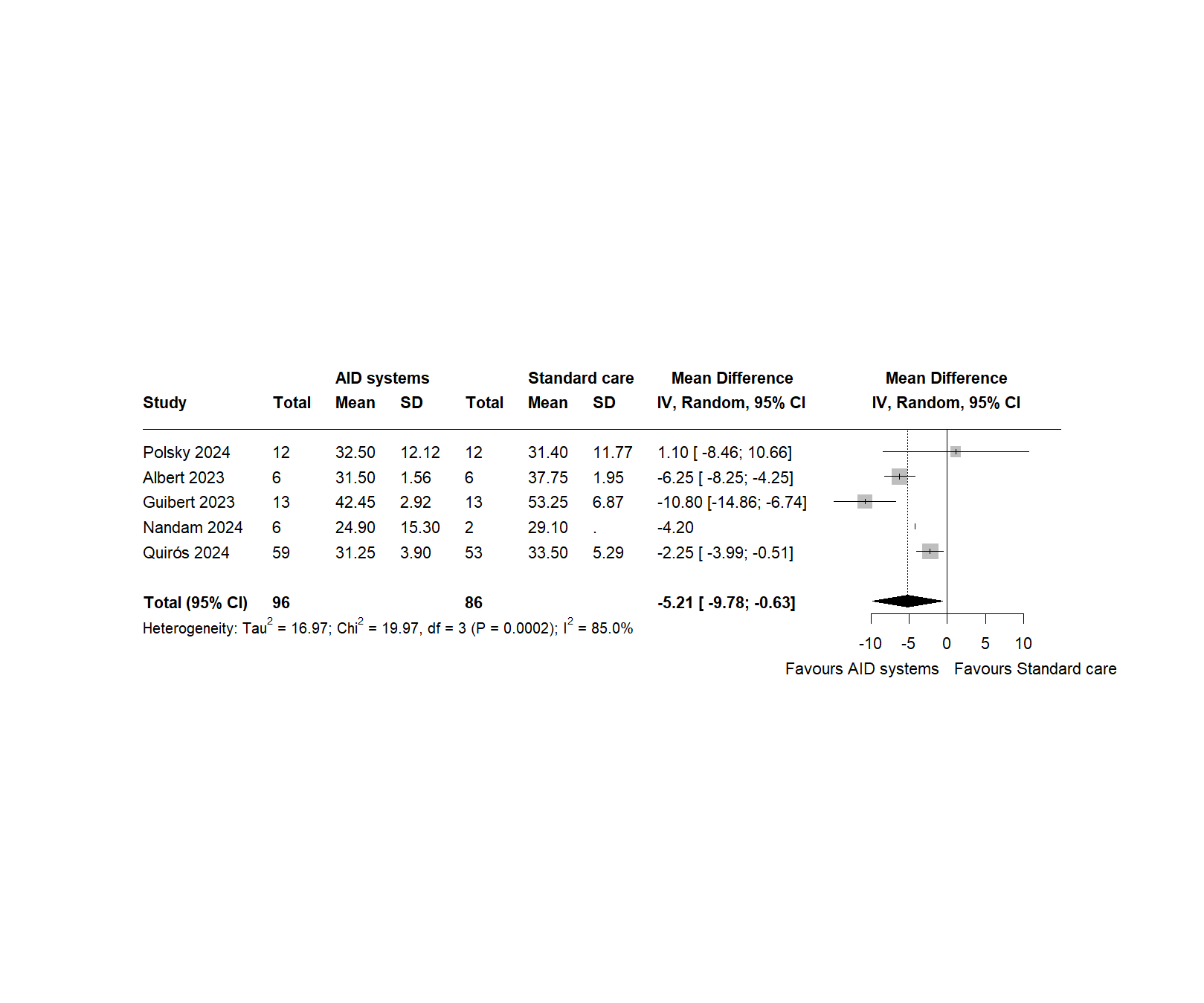


# **Figure S28 - Forest plot of time spent in hypoglycaemia <63 mg/dl during the 1^st^ trimester. Overall effect.**


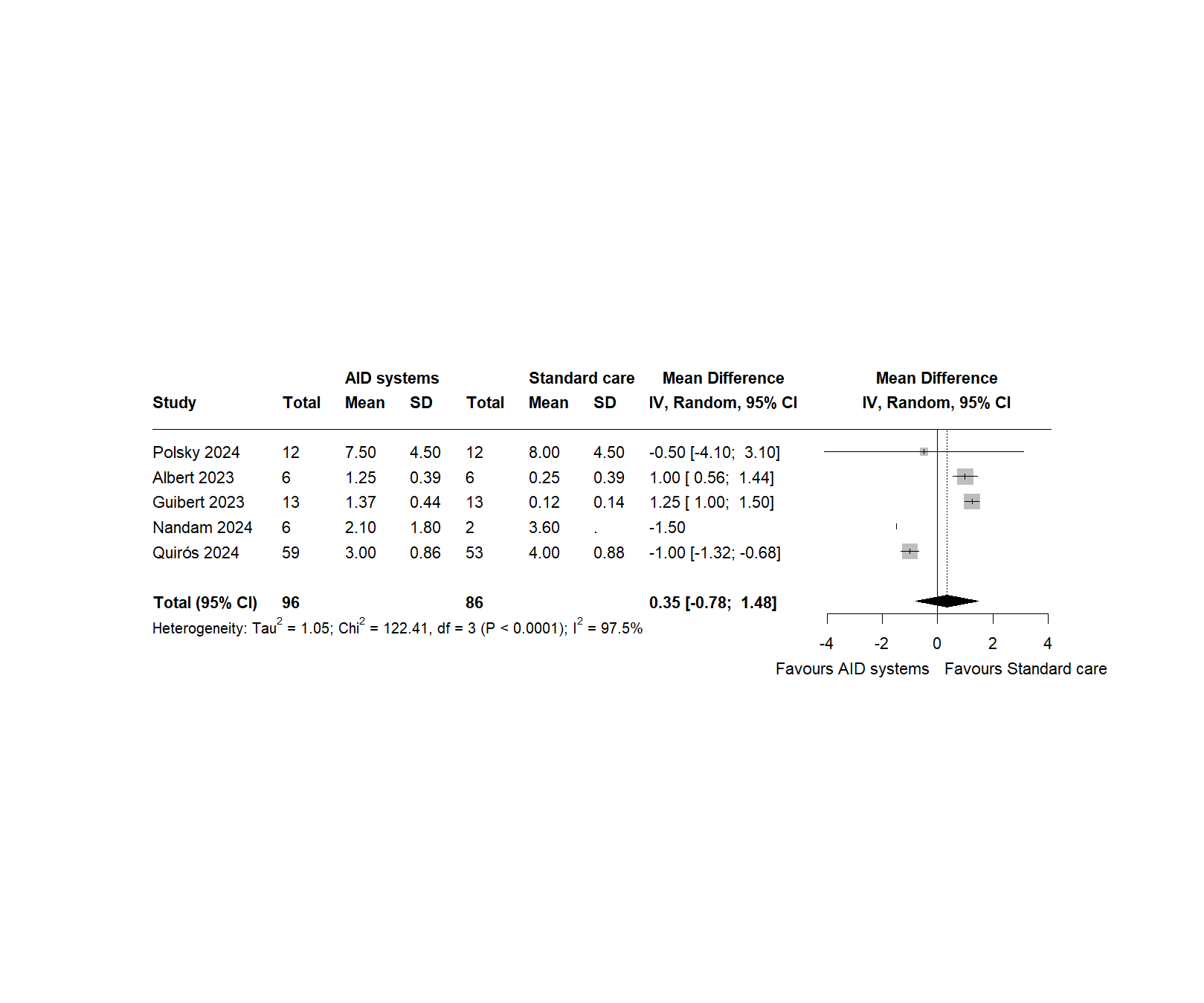


# **Figure S29 - Forest plot of changes in HbA1c (%) during the 1^st^ trimester. Overall effect.**


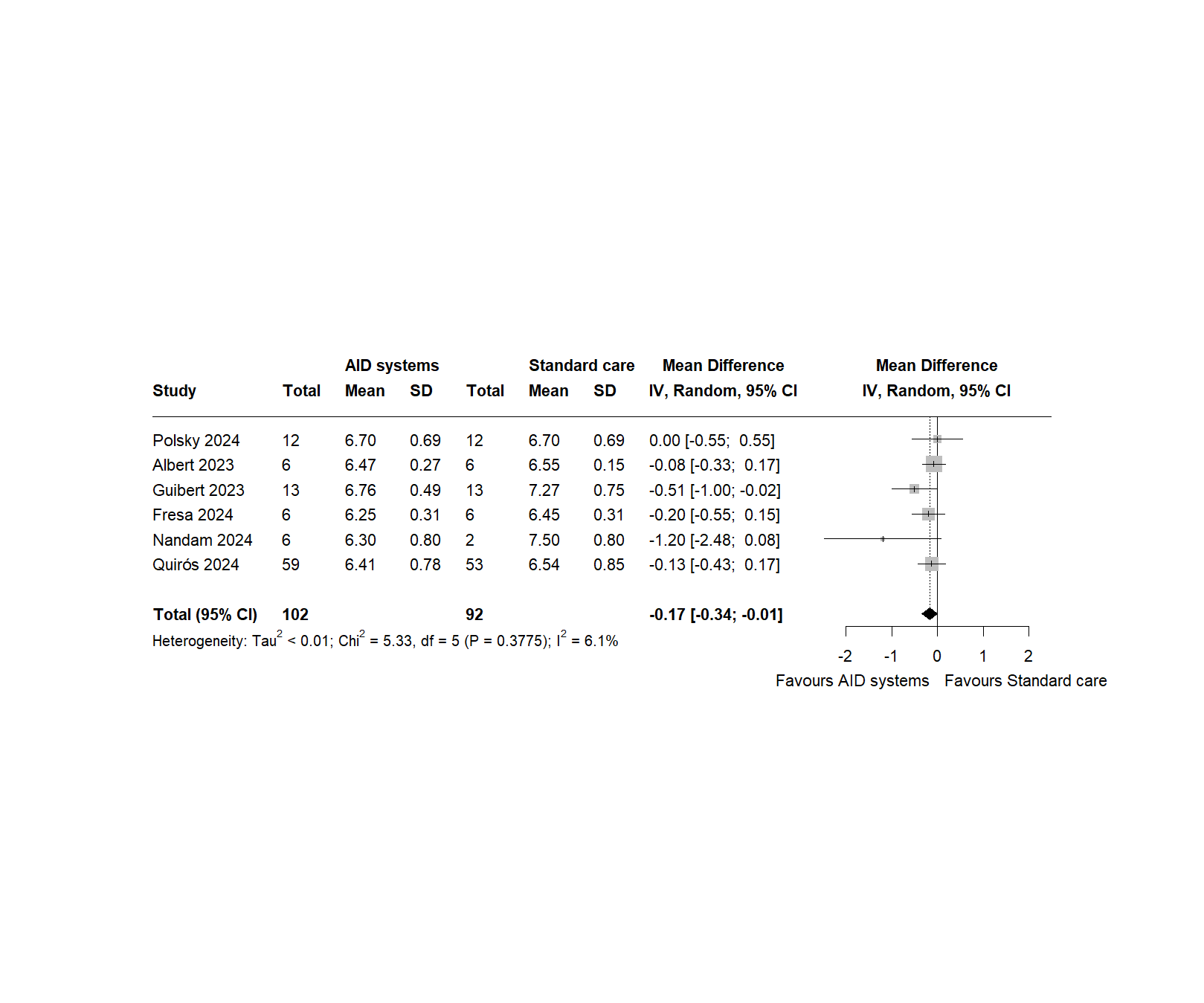


# **Figure S30 - Forest plot of glycaemic variability (CV, %) during the 1^st^ trimester. Overall effect.**


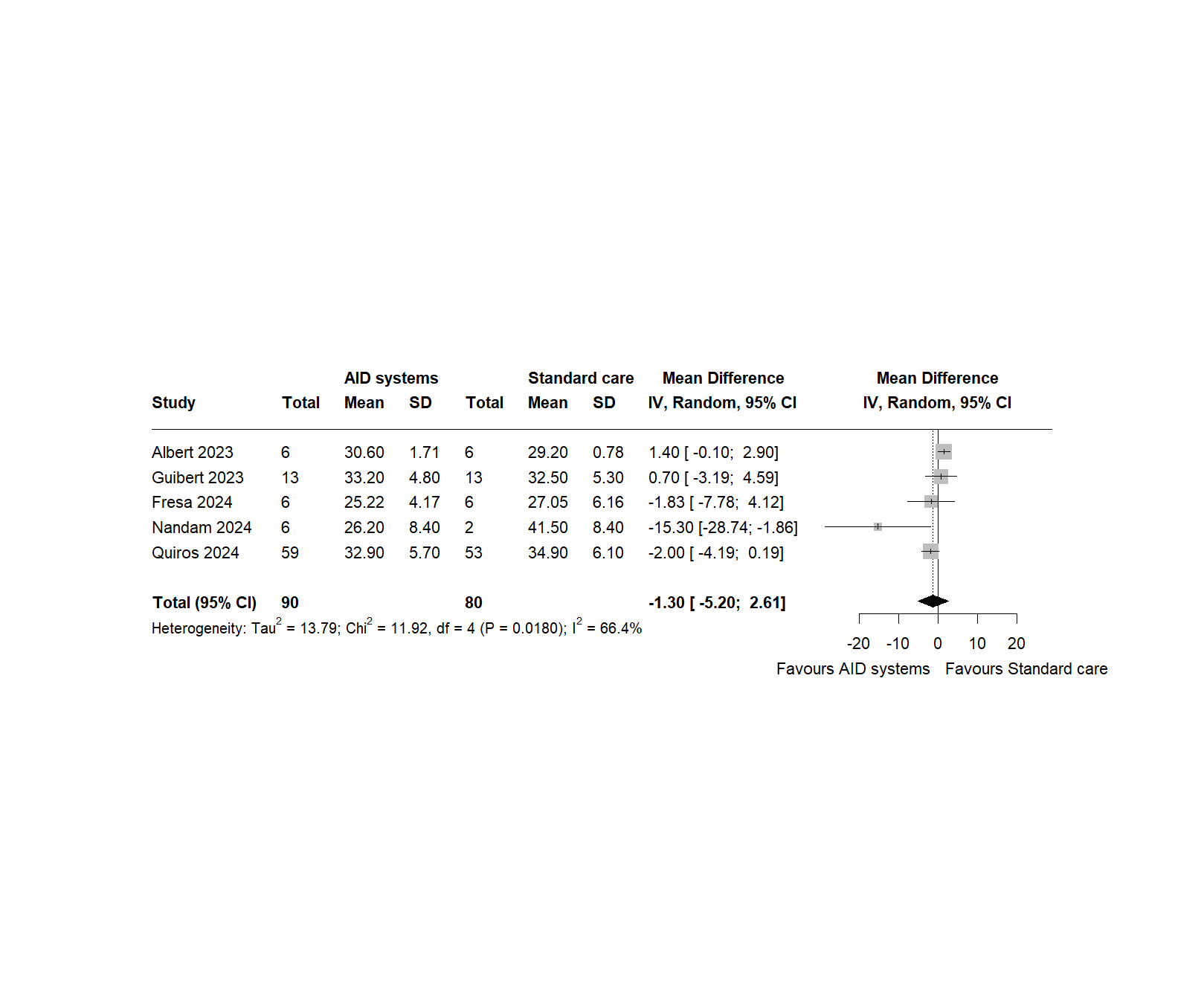


# **Figure S31 - Forest plot of time spent in range 63-140 mg/dl during the 2^nd^ trimester. Overall effect.**


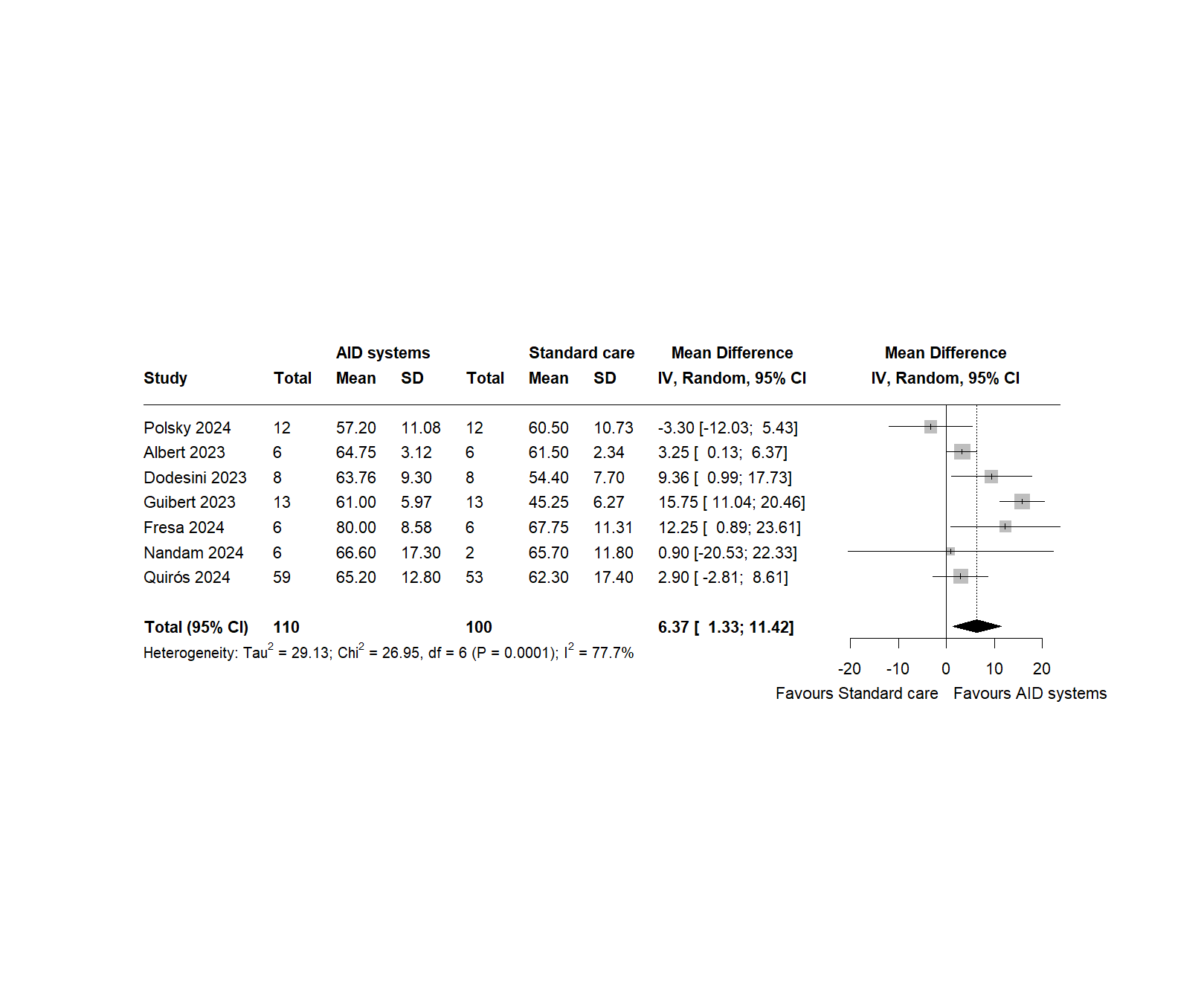


# **Figure S32 - Forest plot of time spent in hypoglycaemia <63 mg/dl during the 2^nd^ trimester. Overall effect.**


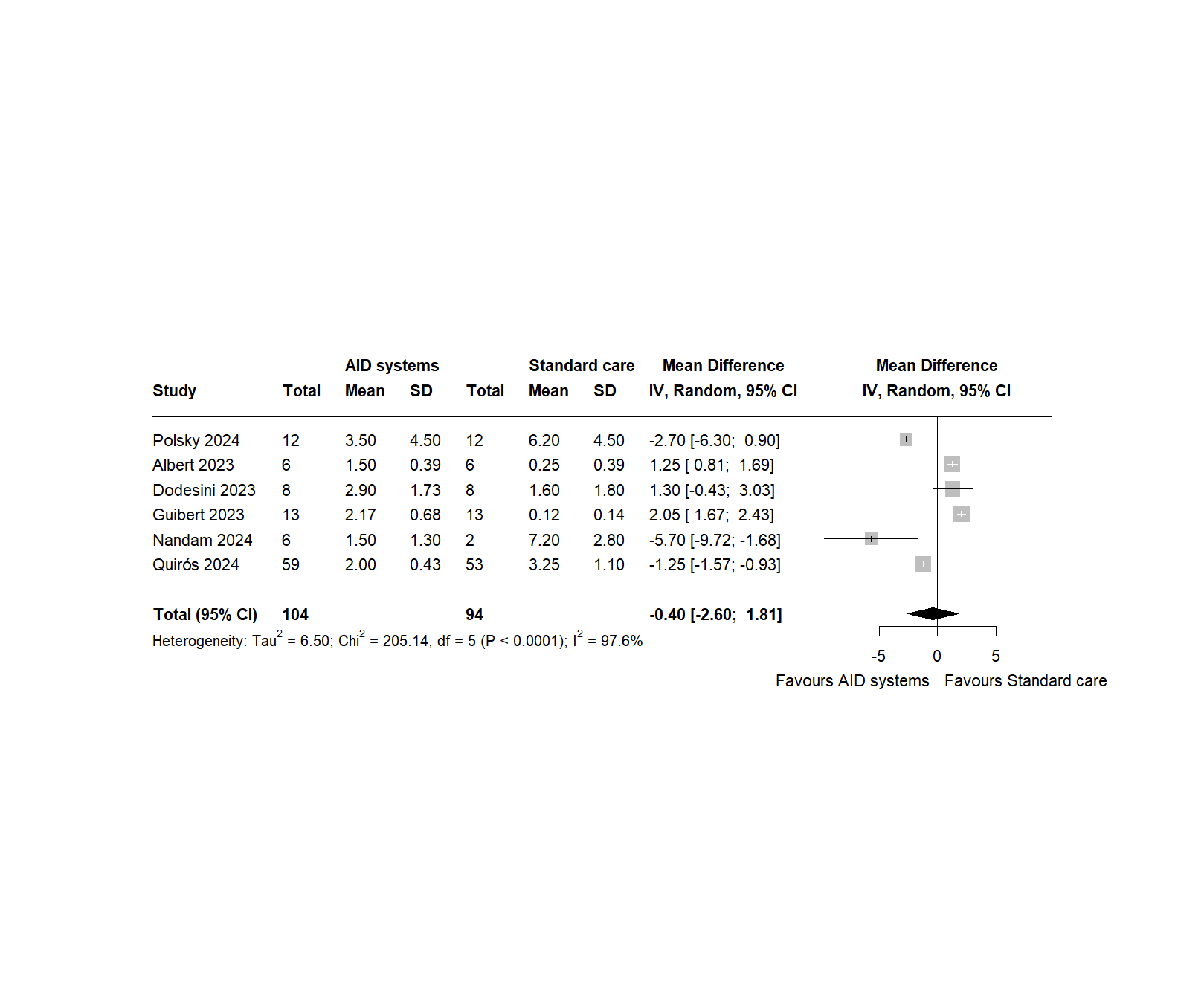


# **Figure S33 - Forest plot of time spent in hyperglycaemia >140 mg/dl during the 2^nd^ trimester. Overall effect.**


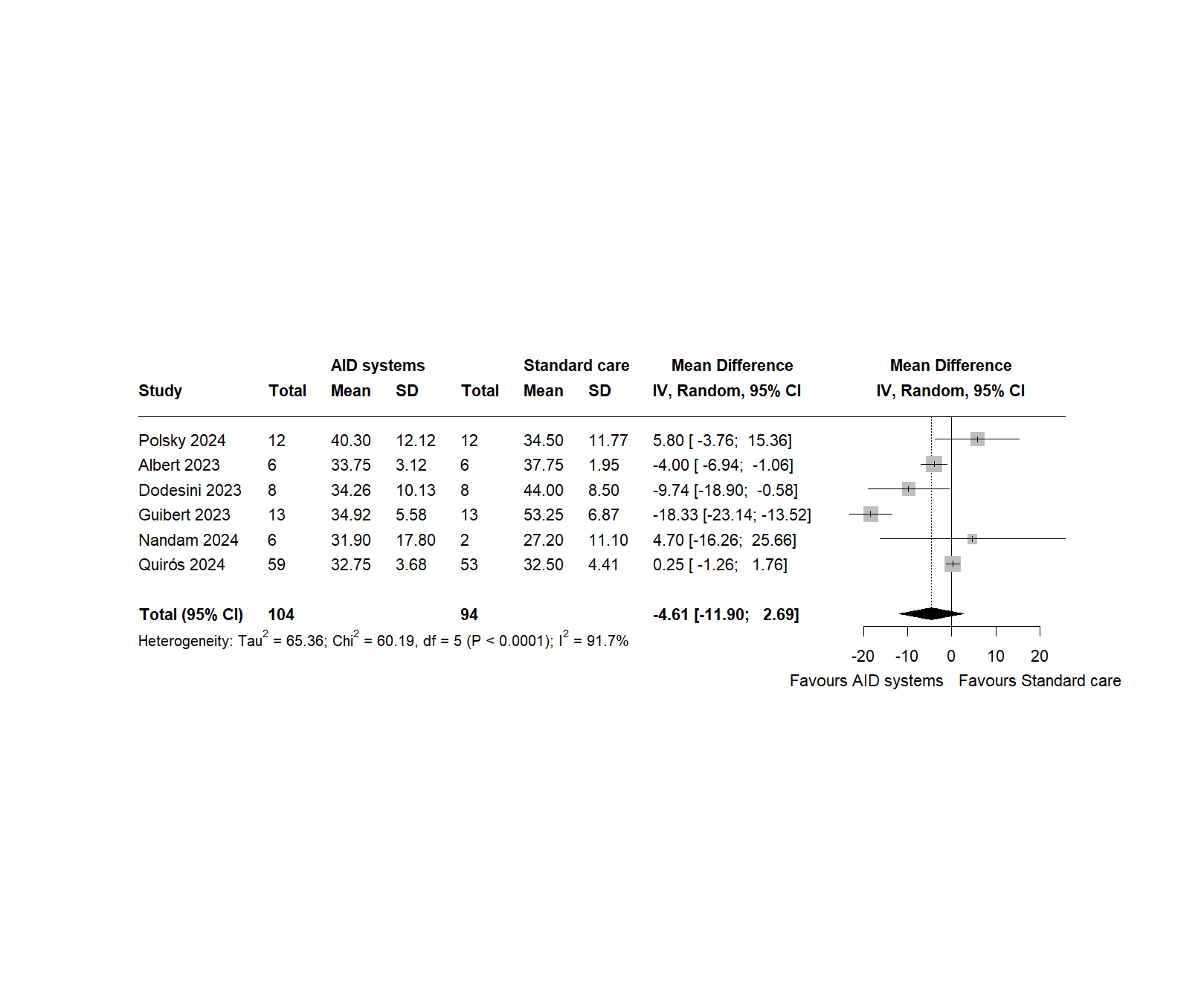


# **Figure S34 - Forest plot of changes in HbA1c (%) during the 2^nd^ trimester. Overall effect.**


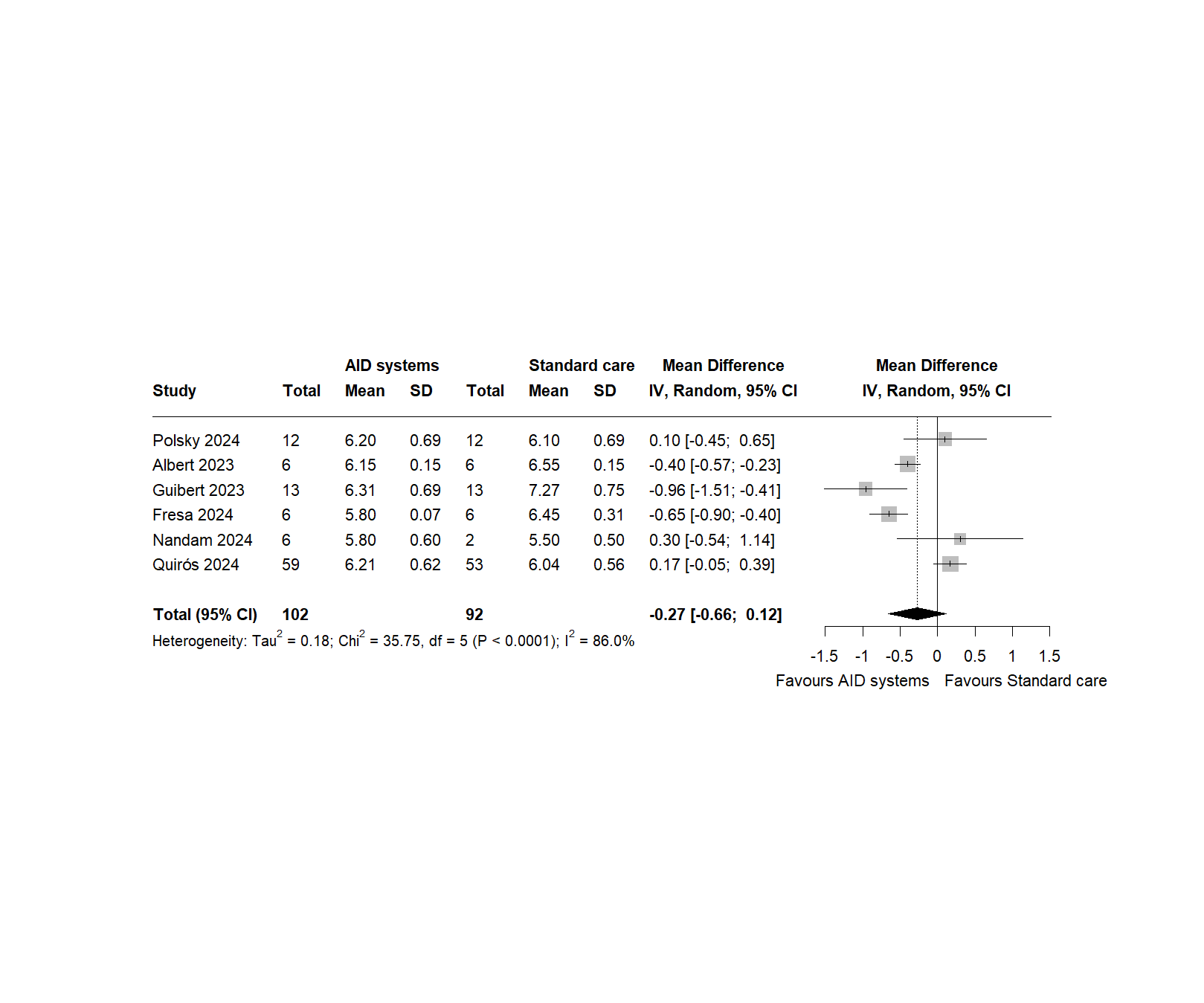


# **Figure S35 - Forest plot of glycaemic variability (CV, %) during the 2^nd^ trimester. Overall effect.**


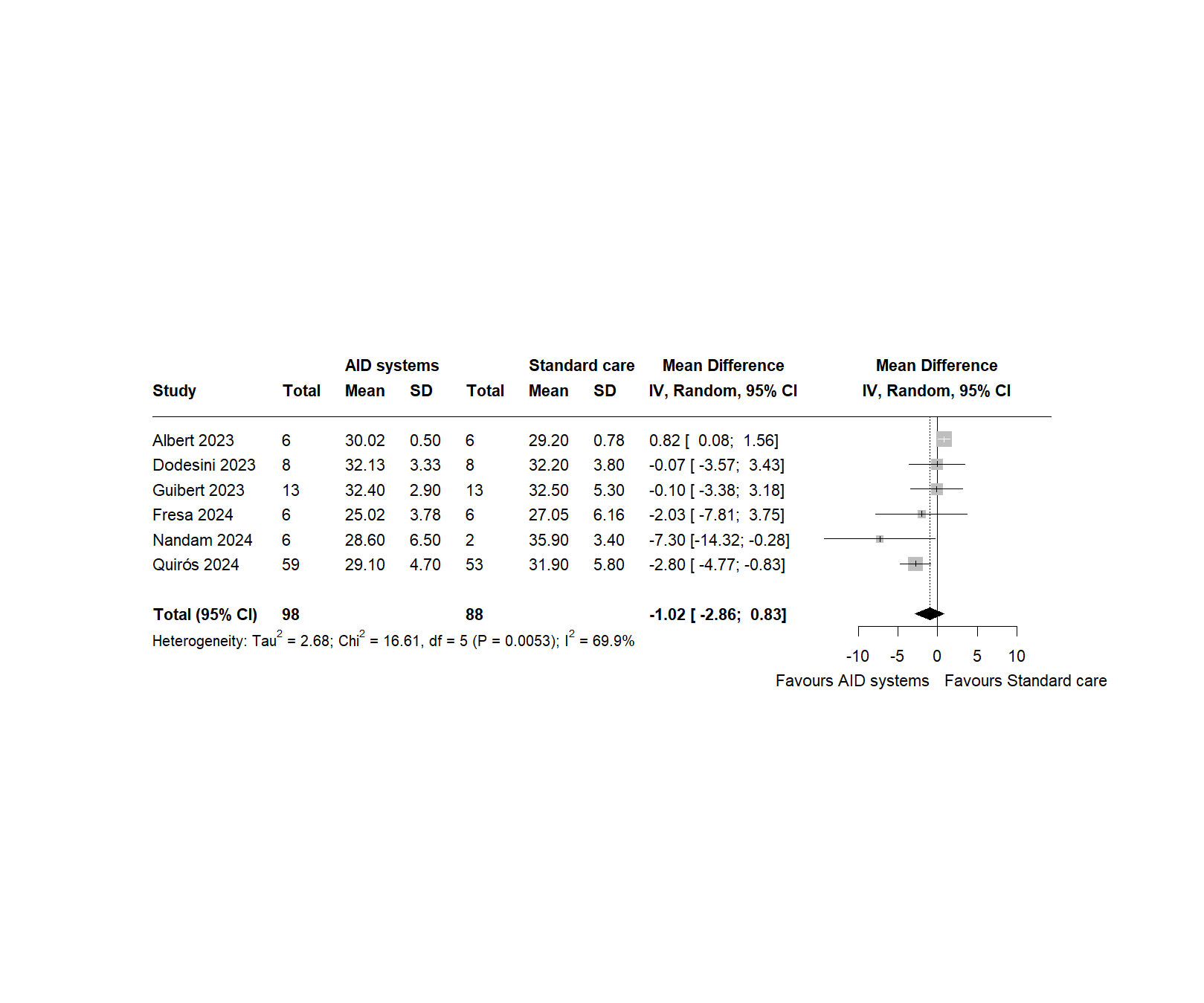


# **Figure S36 - Forest plot of glycaemic variability (CV, %) during the 3^rd^ trimester. Overall effect.**


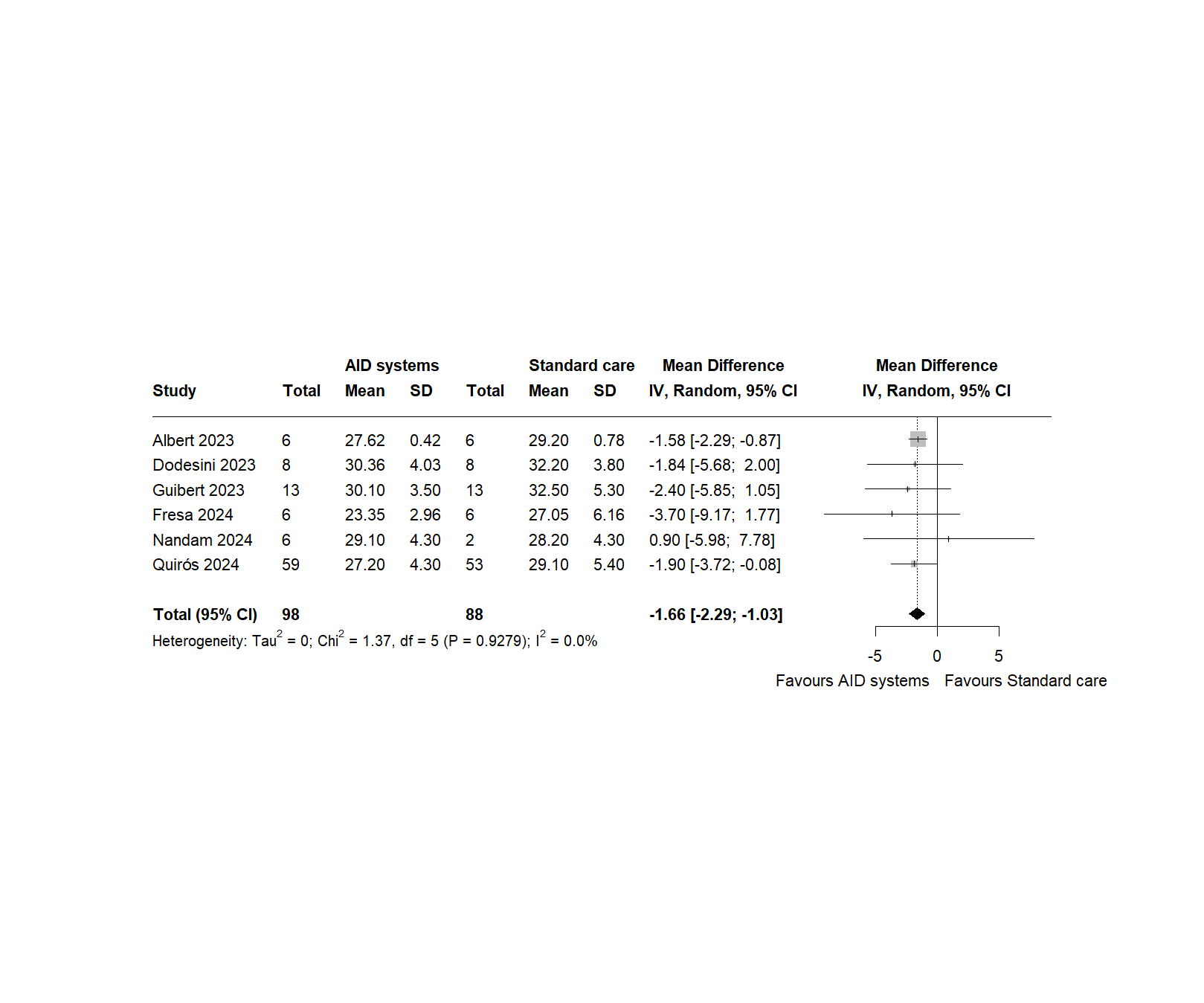


# **Figure S37 - Forest plot of time spent in range 63-140 mg/dl during the 3^rd^ trimester. Overall effect.**


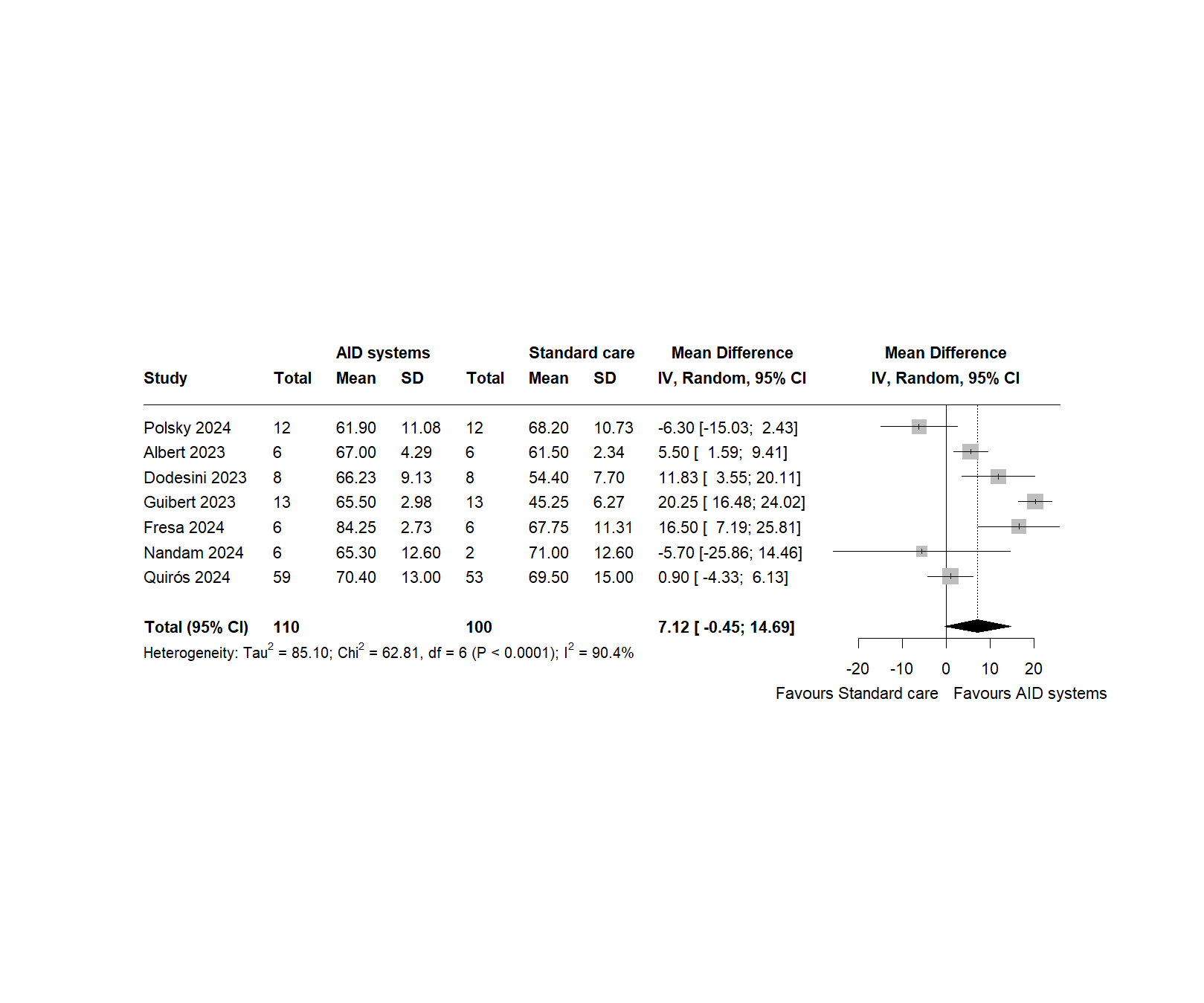


# **Figure S38 - Forest plot of time spent in hypoglycaemia <63 mg/dl during the 3^rd^ trimester. Overall effect.**


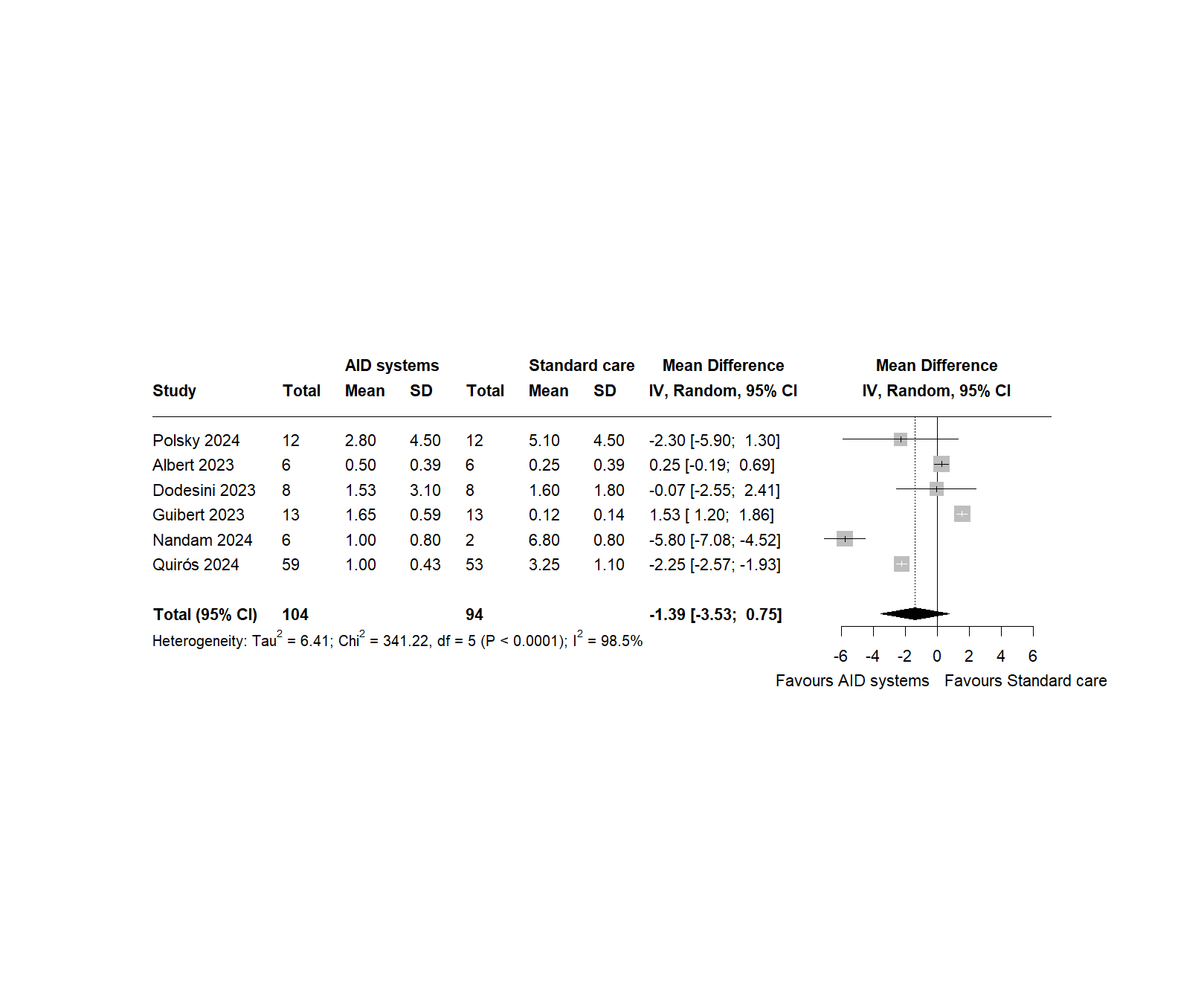


# **Figure S39 - Forest plot of time spent in hyperglycaemia >140 mg/dl during the 3^rd^ trimester. Overall effect.**


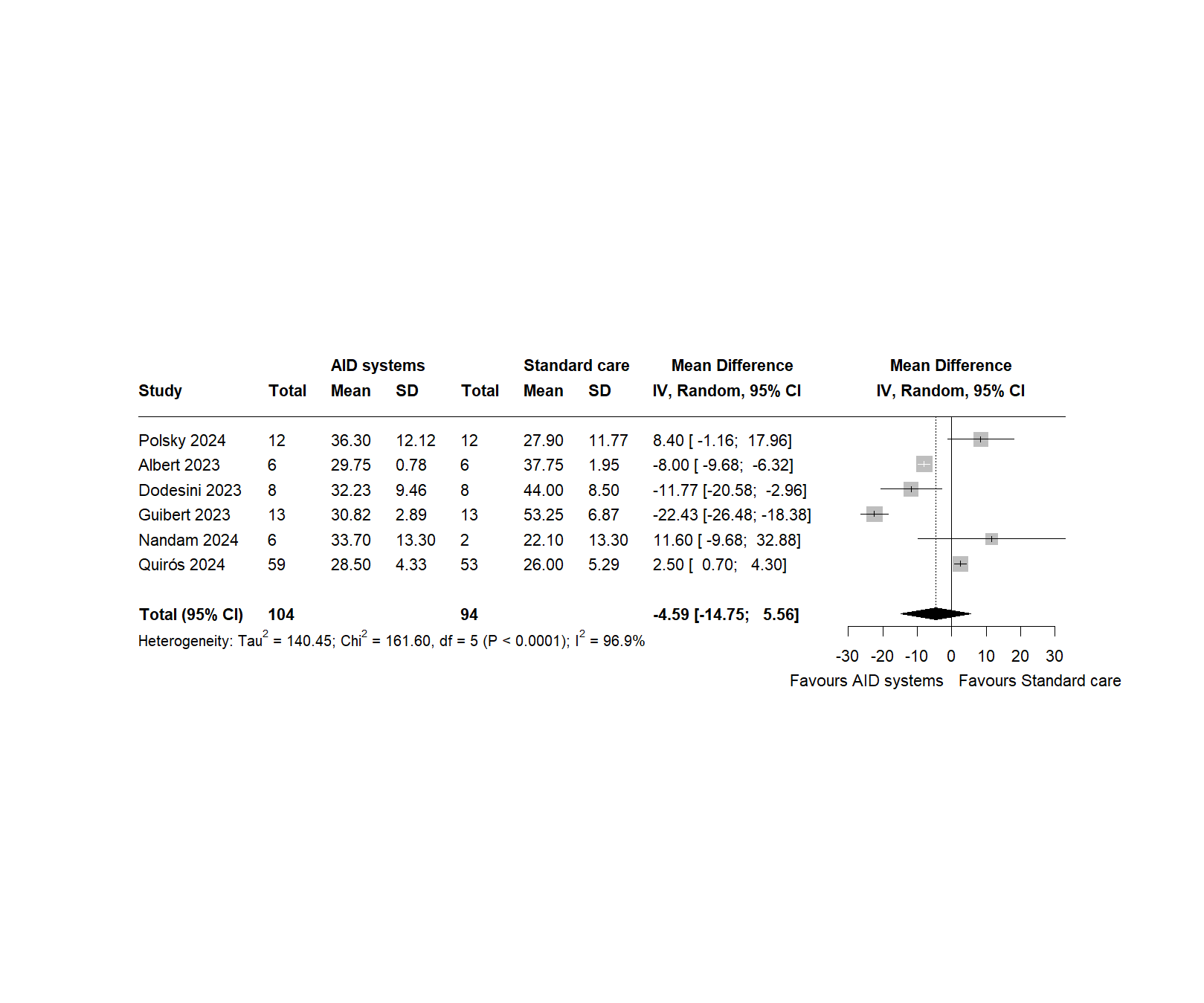


# **Figure S40 - Forest plot of changes in HbA1c (%) during the 3^rd^ trimester. Overall effect.**


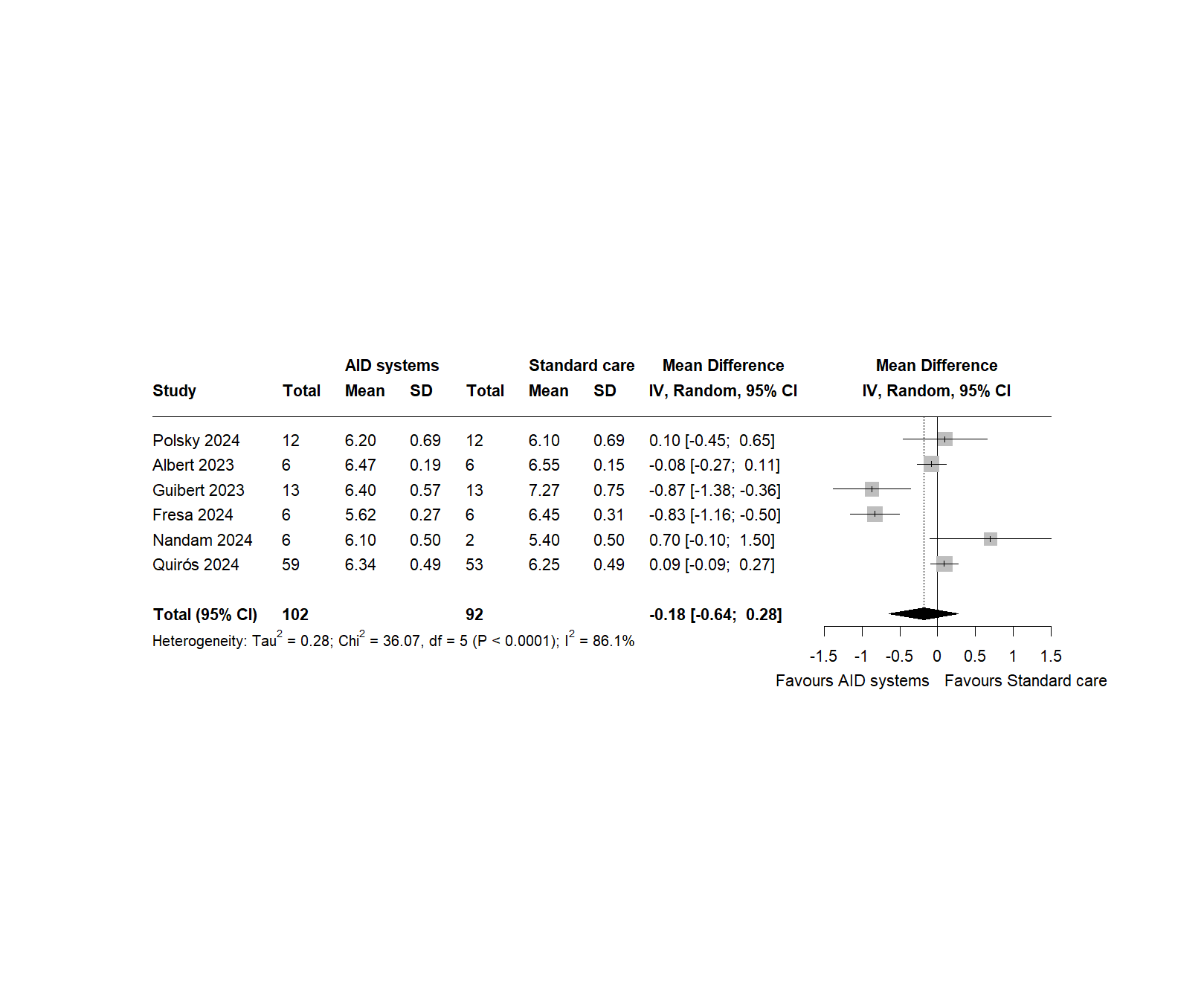

Supplement: Supplementary file 1 — Supplementary file1 (DOCX 1702 KB) [file 592_2025_2446_MOESM1_ESM.docx]
